# A Genome-Wide Search for Gene-Environment Effects in Isolated Cleft Lip with or without Cleft Palate Triads Points to an Interaction between Maternal Periconceptional Vitamin Use and Variants in *ESRRG*

**DOI:** 10.3389/fgene.2018.00060

**Published:** 2018-02-26

**Authors:** Øystein A. Haaland, Rolv T. Lie, Julia Romanowska, Miriam Gjerdevik, Håkon K. Gjessing, Astanand Jugessur

**Affiliations:** ^1^Department of Global Public Health and Primary Care, University of Bergen, Bergen, Norway; ^2^Centre for Fertility and Health, Norwegian Institute of Public Health, Oslo, Norway; ^3^Computational Biology Unit, University of Bergen, Bergen, Norway; ^4^Department of Genetics and Bioinformatics, Norwegian Institute of Public Health, Oslo, Norway

**Keywords:** gene-environment interaction, GWAS, case-parent triad, orofacial cleft, cleft lip with or without cleft palate, birth defects, genetic epidemiology, Haplin

## Abstract

**Background:** It is widely accepted that cleft lip with or without cleft palate (CL/P) results from the complex interplay between multiple genetic and environmental factors. However, a robust investigation of these gene-environment (GxE) interactions at a genome-wide level is still lacking for isolated CL/P.

**Materials and Methods:** We used our R-package Haplin to perform a genome-wide search for GxE effects in isolated CL/P. From a previously published GWAS, genotypes and information on maternal periconceptional cigarette smoking, alcohol intake, and vitamin use were available on 1908 isolated CL/P triads of predominantly European or Asian ancestry. A GxE effect is present if the relative risk estimates for gene-effects in the offspring are different across exposure strata. We tested this using the relative risk ratio (RRR). Besides analyzing all ethnicities combined (“pooled analysis”), separate analyses were conducted on Europeans and Asians to investigate ethnicity-specific effects. To control for multiple testing, *q*-values were calculated from the *p*-values.

**Results:** We identified significant GxVitamin interactions with three SNPs in “Estrogen-related receptor gamma” (*ESRRG*) in the pooled analysis. The RRRs (95% confidence intervals) were 0.56 (0.45–0.69) with rs1339221 (*q* = 0.011), 0.57 (0.46–0.70) with rs11117745 (*q* = 0.011), and 0.62 (0.50–0.76) with rs2099557 (*q* = 0.037). The associations were stronger when these SNPs were analyzed as haplotypes composed of two-SNP and three-SNP combinations. The strongest effect was with the “t-t-t” haplotype of the rs1339221-rs11117745-rs2099557 combination [RRR = 0.50 (0.40–0.64)], suggesting that the effects observed with the other SNP combinations, including those in the single-SNP analyses, were mainly driven by this haplotype. Although there were potential GxVitamin effects with rs17734557 and rs1316471 and GxAlcohol effects with rs9653456 and rs921876 in the European sample, respectively, none of the SNPs was located in or near genes with strong links to orofacial clefts. GxAlcohol and GxSmoke effects were not assessed in the Asian sample because of a lack of observations for these exposures.

**Discussion/Conclusion:** We identified significant interactions between vitamin use and variants in *ESRRG* in the pooled analysis. These GxE effects are novel and warrant further investigations to elucidate their roles in orofacial clefting. If validated, they could provide prospects for exploring the impact of estrogens and vitamins on clefting, with potential translational applications.

## Introduction

With a prevalence of 3.4–22.9 per 10,000 live births (Mossey and Castilla, 2003), cleft lip with or without cleft palate (CL/P) ranks among the most common birth defects in humans. It is widely accepted that CL/P results from the complex interplay between multiple genetic and environmental factors (GxE), but only recently have data and practical approaches become available to enable an investigation of these effects at a genome-wide level. Identifying GxE interactions may not only provide important insights into the etiology of orofacial clefts, but may also be important from a public health perspective because of the potential for interventions on environmental risk factors alone, particularly in genetically more susceptible subgroups of the population. This rationale has long been demonstrated in animal models (Millicovsky and Johnston, 1981a,b; Juriloff, 2002), but the evidence in humans is less conclusive. Just as some murine strains are more susceptible to external teratogens, human fetuses harboring high-risk alleles may also be more sensitive to particular environmental risk factors, and identifying those that interact with genetic risk variants may lead to important inroads in our understanding of the causes of CL/P.

Over the years, a growing list of maternal exposures has been reported to influence the risk of isolated CL/P. In particular, cigarette smoking (Zeiger and Beaty, 2002; Little et al., 2004; Lie et al., 2008), alcohol consumption (DeRoo et al., 2008), folic acid and other B-complex vitamin supplementation (Hayes, 2002; Munger, 2002; Munger et al., 2004; Wilcox et al., 2007), and anti-folate medication (Hernández-Díaz et al., 2000; Holmes et al., 2001) have been among the most widely studied exposures. Associations with other environmental risk factors have also been reported (reviewed in Dixon et al., 2011), but these have been less consistent across studies and no consensus has yet emerged on the harmful effects of these exposures.

A wide variety of study designs have been used to study GxE effects in orofacial clefts, one of which involves treating a case-parent triad as the unit of analysis. This family-based “triad design” handles bias due to population stratification by using non-transmitted parental alleles as controls, to be compared with the alleles transmitted to the case child (Gjessing and Lie, 2006). We and others have used the triad design to investigate GxE effects in orofacial clefts (Jugessur et al., 2003; Shi et al., 2007; Wu et al., 2010). In terms of statistical power, a unit comprising one case and one control provides approximately the same power as a complete triad when studying single-SNP associations (Schaid, 2002). For haplotype reconstruction, however, the triad design offers the additional advantage of allowing haplotypes to be deduced from the family structure (Gjessing and Lie, 2006).

A handful of genome-wide association studies (GWAS) have now been published on orofacial clefts (reviewed in Dixon et al., 2011; Mangold et al., 2011; Rahimov et al., 2012; Beaty et al., 2016), offering unprecedented opportunities for investigating GxE effects at a genome-wide level. Using data on case-parent triads from a previously published GWAS (Beaty et al., 2010), Wu et al. (2014) and Beaty et al. (2011) screened for GxE effects in one category of orofacial clefts: isolated cleft palate only (CPO). Here, we used the same GWAS dataset to search for GxE effects in the larger category of isolated CL/P.

## Materials and methods

### Study participants

**Figure 1:**
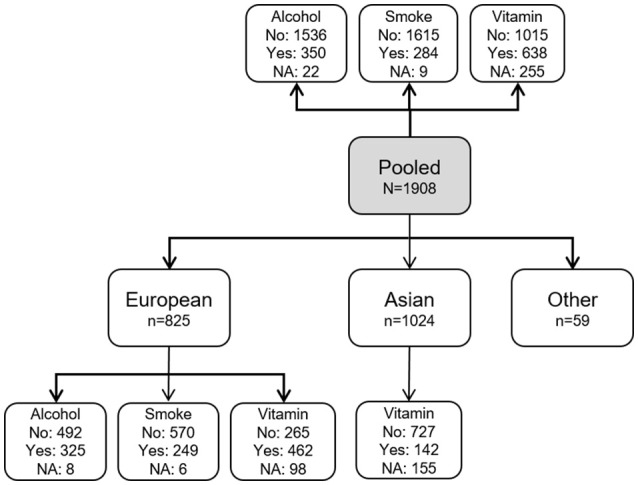
Distribution of families according to ethnicity and maternal exposure status. The gray box shows the pooled sample consisting of all ethnicities (*N* = 1,908). White boxes show the number of families according to ethnicity and exposure (yes: exposed; no: unexposed; NA: data not available). Note that we did not consider GxSmoke or GxAlcohol analyses in the Asian sample because of a very low number of observations for these exposures.

Participants in this study stem from an international cleft collaboration involving seven Asian and six European/US populations. Characteristics of the study populations and details of the GWAS have been provided in Beaty et al. (2010). Briefly, genotyping was performed on an Illumina Human610-Quad® platform and genotypes for 589,945 SNPS were deposited in the Database of Genotypes and Phenotypes (dbGaP; http://www.ncbi.nlm.nih.gov/gap) under study accession ID phs000094.v1.p1. Besides genotypes, information was also available on maternal cigarette smoking, alcohol intake, and vitamin use in the periconceptional period (i.e., 3 months prior to conception through the first trimester of pregnancy). Interviews and questionnaires were used to assess maternal exposures and the data were coded as simple yes/no responses for cigarette smoking, any reported alcohol consumption, and any use of vitamin supplements. For a detailed description of these maternal exposures, see the recent work on isolated CPO by Wu et al. (2014) and the study outline at dbGAP under study accession ID phs000094.v1.p1. Quality control was performed using the approach outlined in Haaland et al. (2017). The number of SNPs before and after the pruning process is provided in Table 1. Figure 1 shows the distribution of triads by ethnicity and environmental exposure after quality control was performed. Note that we did not analyze GxSmoke or GxAlcohol in the Asian sample because very few of the mothers reported smoking cigarettes or drinking alcohol in the periconceptional period. The final version of the dataset included genotypes for 1,908 nuclear families; 825 were of European ancestry, 1,024 of Asian descent, and 59 of other ethnicities. Table 2 provides an overview of triad completeness and ethnicity. Among the 1,908 nuclear families, 1,594 were complete mother-father-child triads and 314 were parent–child dyads only.

**Table 1:** Number of SNPs before and after pruning.

**Criteria**	**Number**
Total no. of SNPs before pruning	569,244
Failed HWE test	173,955
Failed missingness test	1,934
Failed SNP frequency test	61,167
Mendelian errors detected	349
Remaining SNPs after pruning^a^	341,191

a *Remaining SNPs refer to those without deviations from Hardy-Weinberg equilibrium (HWE) (p < 0.001), and those having less than 5% missed calls, minor allele frequencies >5%, and Mendelian errors <1%. Note that because some SNPs may fail several criteria, the number of remaining SNPs is not equal to the total number of SNPs minus those removed in the pruning process*.

**Table 2:** Triad completeness by ethnicity^a^.

**Ethnicity**	**Complete triads^c^**		**Incomplete triads^d^**		**Total**	
	**Individuals**	**Families**	**Individuals**	**Families**	**Individuals**	**Families**
European	2,024	670	310	155	2,334	825
Asian	2,670	890	268	134	2,938	1,024
Other^b^	102	34	50	25	152	59
Pooled	4,796	1,594	628	314	5,424	1,908

a *In total, 317 individuals had genotype call rates <10% and were removed from the analyses. Columns show the number of remaining families and individuals*.

b *Analyses were not conducted for this group because of small numbers*.

c *Some families had more than one offspring*.

d *These are parent-offspring dyads*.

### Statistical analysis

We are continuously extending a comprehensive R-package, Haplin, for analyzing different constellations of family-based data (Gjessing and Lie, 2006). Haplin uses maximum likelihood to estimate the relative risk (RR), *p*-value, and 95% confidence intervals for a given risk allele or haplotype. To identify GxE effects, a Wald test is used to test whether RR estimates for gene-effects in the offspring are significantly different across strata of exposed and unexposed mothers, as described in our previous works (Skare et al., 2012; Gjerdevik et al., 2017). A GxE effect is assessed as the ratio of relative risks (RRR). That is, RRR = RR(1)/RR(0), where RR(1) and RR(0) are the relative risks for the offspring of exposed and unexposed mothers, respectively. If RRR ≠ 1, the RR among the exposed is different from the RR among the unexposed, which would indicate a GxE effect. See Haaland et al. (2017) for more details on the calculation of RRR.

Since its inception, Haplin has incorporated haplotype estimation for triads. By using the expectation-maximization (EM) algorithm (Dempster et al., 1977), Haplin reconstructs haplotypes from the multi-SNP data even though phase is not known from the observed markers alone. The EM-algorithm also enables Haplin to account for missing parental genotypes, thus allowing the 314 parent–child dyads in the current dataset to be included in the analyses.

If an offspring was homozygous for the risk allele at a locus, we assumed a multiplicative dose-response model. In other words, the relative risk with two copies of the risk allele was assumed to be simply RR × RR. For each maternal exposure (smoking, alcohol use, vitamin use), we performed separate analyses on Asians (*n* = 1,024), Europeans (*n* = 825), and all participants combined irrespective of their ethnicity (“pooled sample”; *N* = 1,908). The different sets of analyses are illustrated in Figure 1.

Further, we conducted haplotype analyses for SNPs that had very low *q*-values. This was done using the haplinSlide function in Haplin, as described in our previous works on candidate genes for clefts (Jugessur et al., 2008, 2009b, 2012a,b).

Next, we performed statistical power calculations using the hapPowerAsymp function in Haplin. It is based on the asymptotic variance-covariance structure of the Wald test, as described in our recent work (Gjerdevik et al., 2017). The sample sizes used in these calculations reflect those that were available in the current GWAS dataset (Figure 1).

To facilitate the interpretation of the large number of statistical tests being performed simultaneously, we generated Quantile-Quantile (QQ) plots (Wilk and Gnanadesikan, 1968) using the pQQ function in Haplin to screen for significant *p*-values. This was done separately for the analysis of each exposure. To control the false discovery rate, we used the method described by Storey and Tibshirani (2003) where *q*-values are computed from the *p*-values. The function qvalue from the R-package “*q*-value” in the Bioconductor repository [https://www.bioconductor.org/, Gentleman et al., 2004] was used to generate the *q*-values. A low *q*-value indicates a low likelihood that a statistically significant association is a false positive. A *q*-value of 0.1, for instance, corresponds to a false discovery rate of 10%.

A regional plot was generated to map the chromosomal area flanking the most significant SNP (“lead SNP”). This plot provides a visual display of linkage disequilibrium between the lead SNP and other SNPs, along with their positions. It also provides the position of the closest genes and the recombination rates in the region. We generated the plot by modifying an R-script available at http://www.broadinstitute.org/files/shared/diabetes/scandinavs/assocplot.R (Pruim et al., 2010).

The plethora of publicly available data makes it feasible to explore the connections between different entities, e.g., genes and vitamins. Hetionet integrates different sources of information from actively maintained meta-databases and allows the visualization of possible paths between various diseases, genes and compounds (Himmelstein et al., 2017, https://neo4j.het.io/). For example, to explore possible links between a given disease, compounds and gene nodes, Hetionet uses the following databases: Disease Ontology (Kibbe et al., 2015), Drug Bank (Law et al., 2014), and Entrez Gene (Maglott et al., 2005). Furthermore, Hetionet can also integrate information from the Human Interactome Database (Rolland et al., 2014), CMap (https://clue.io/) or Bgee (Bastian et al., 2008) to identify potential gene-gene interactions, compound-gene interactions and gene expression in specific tissue, respectively. We queried Hetionet to visualize the relationship between genes and maternal exposures for the cleft lip phenotype. The complete query and output codes are provided in the online Supplementary Text 1.

### Ethics approvals

Ethics approvals for the International Cleft Consortium were obtained from the respective institutional review boards of the participating sites. The consortium was formed in 2007 and each participating institution approved research protocols for the recruitment of case-parent triads from 13 individual sites. All participants have granted their written informed consents. The participating sites included institutions in the US (Johns Hopkins University; University of Iowa; Utah State University; National Institute of Environmental Health Sciences (NIEHS); University of Pittsburgh), Denmark (University of Southern Denmark), Norway (University of Bergen), China (Peking University Health Science Center; Wuhan University; Peking Union Medical College; West China School of Stomatology, Sichuan University; School of Stomatology, Beijing University), Korea (Yonsei University), Taiwan (Chang Gung Memorial Hospital), and Singapore (KK Women's & Children's Hospital; National University of Singapore). For more details on the recruitment sites, the research approvals and protocols, see the online “Supplementary Note” of the original publication (Beaty et al., 2010), as well as the study outline at dbGAP (https://www.ncbi.nlm.nih.gov/gap) under study accession number phs000094.v1.p1.

## Results

**Figure 2:**
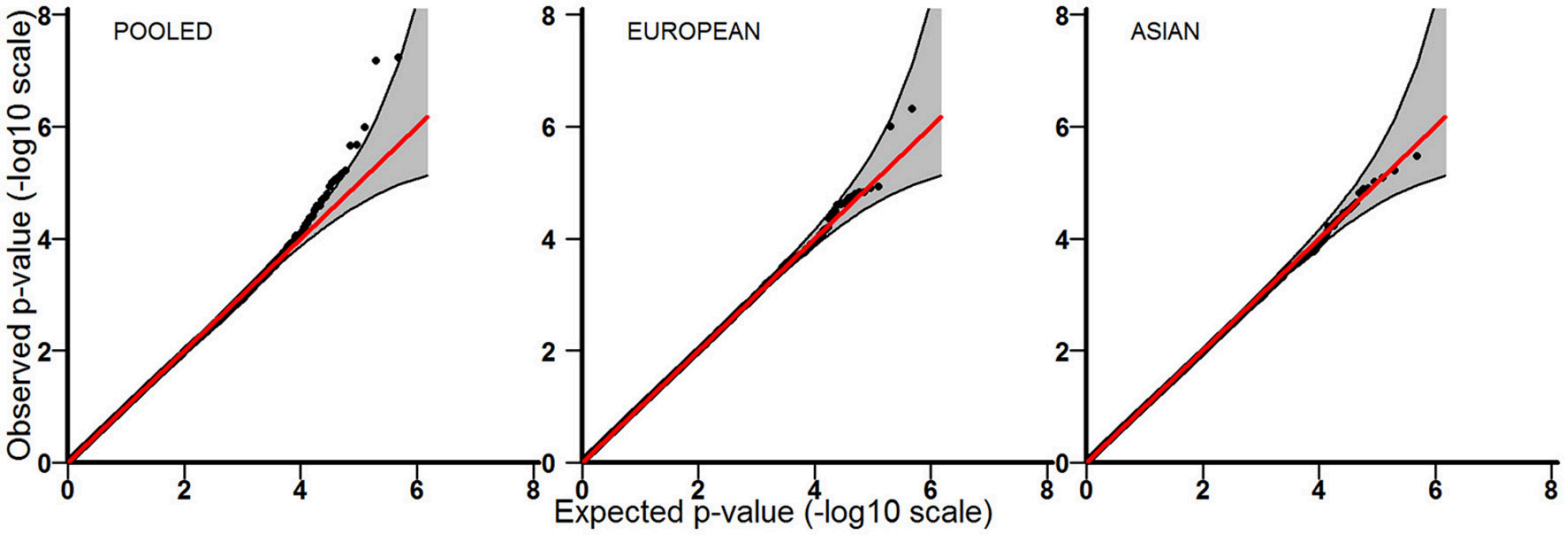
QQ-plots for the GxVitamin analyses in the pooled, European and Asian samples.

**Figure 3:**
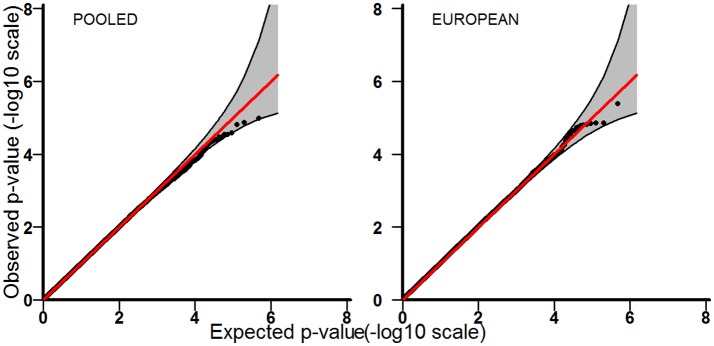
QQ-plots for the GxSmoke analyses in the pooled and European samples.

**Figure 4:**
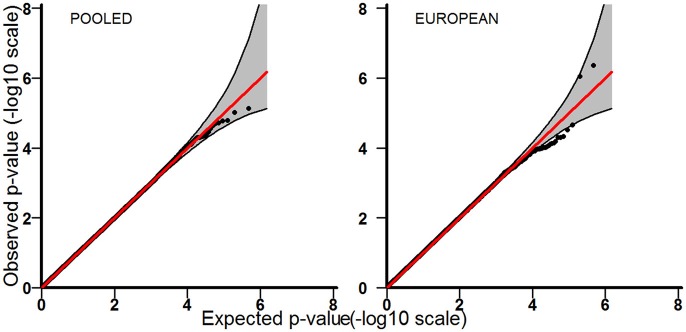
QQ-plots for the GxAlcohol analyses in the pooled and European samples.

**Figure 5:**
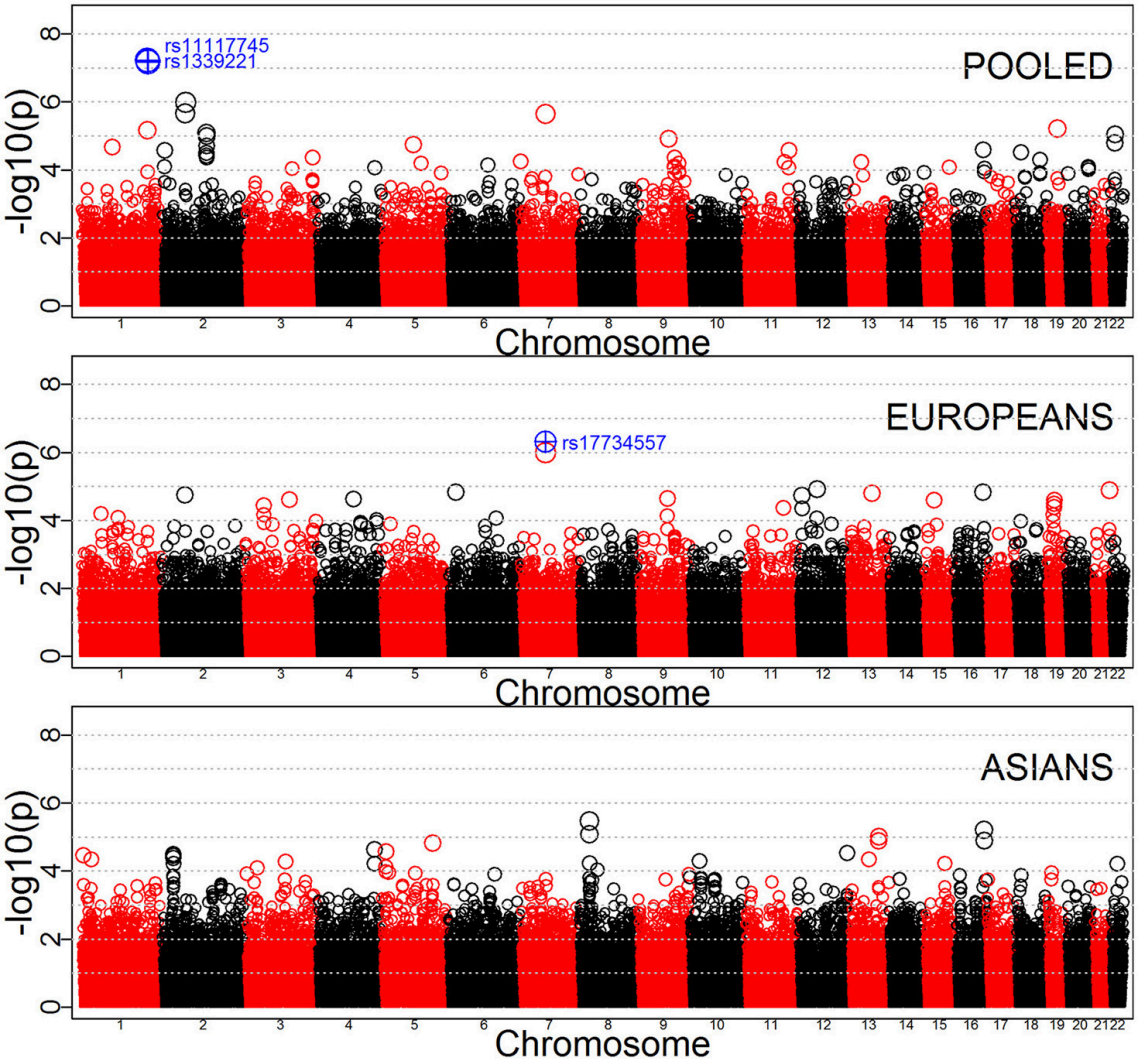
Manhattan plots for GxVitamin effects in the pooled, Europeans-only and Asians-only analyses. SNPs with *p*-values below 10^−6^ are shown in blue.

**Figure 6:**
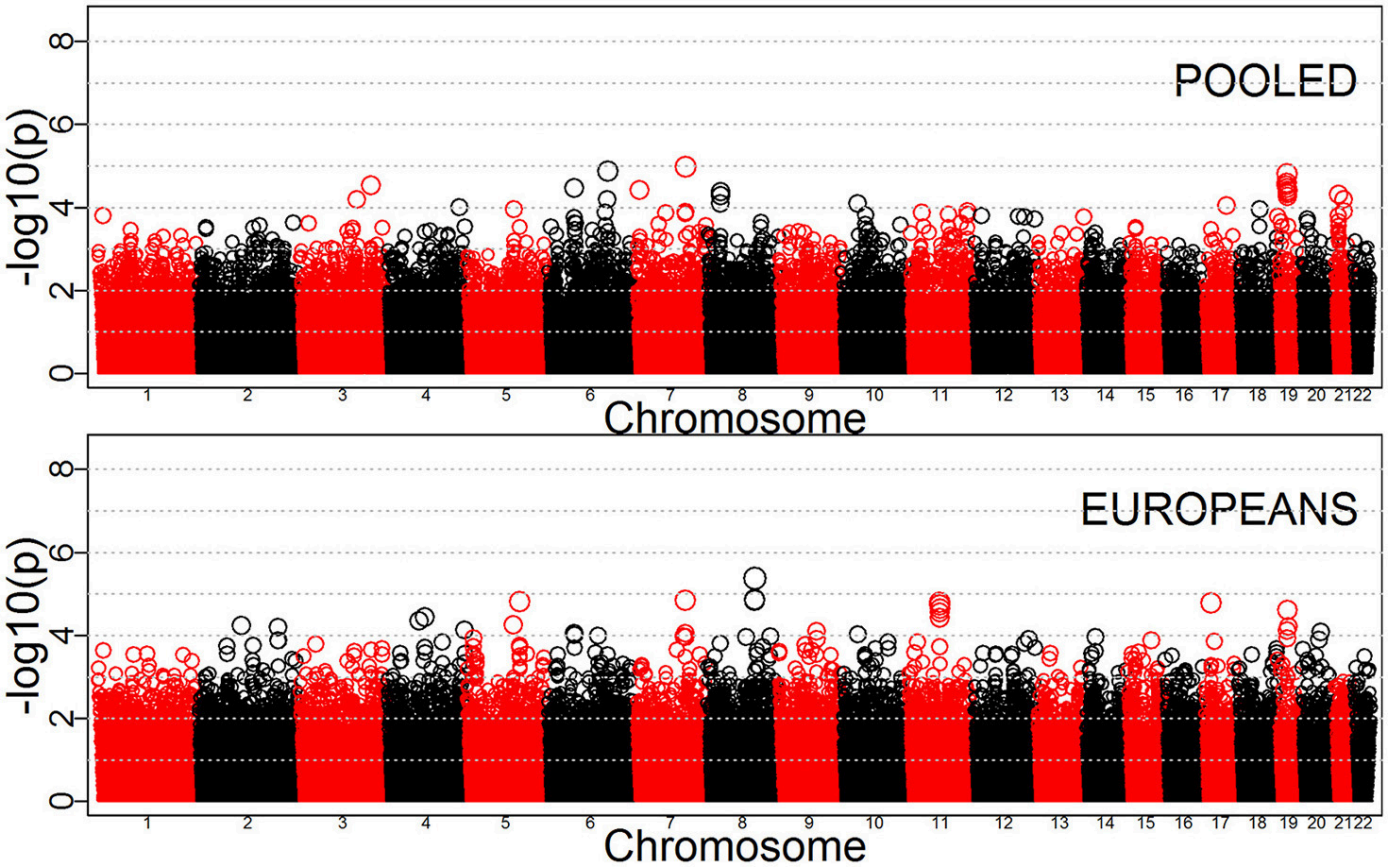
Manhattan plots for GxSmoke effects in the pooled and Europeans-only analyses.

**Figure 7:**
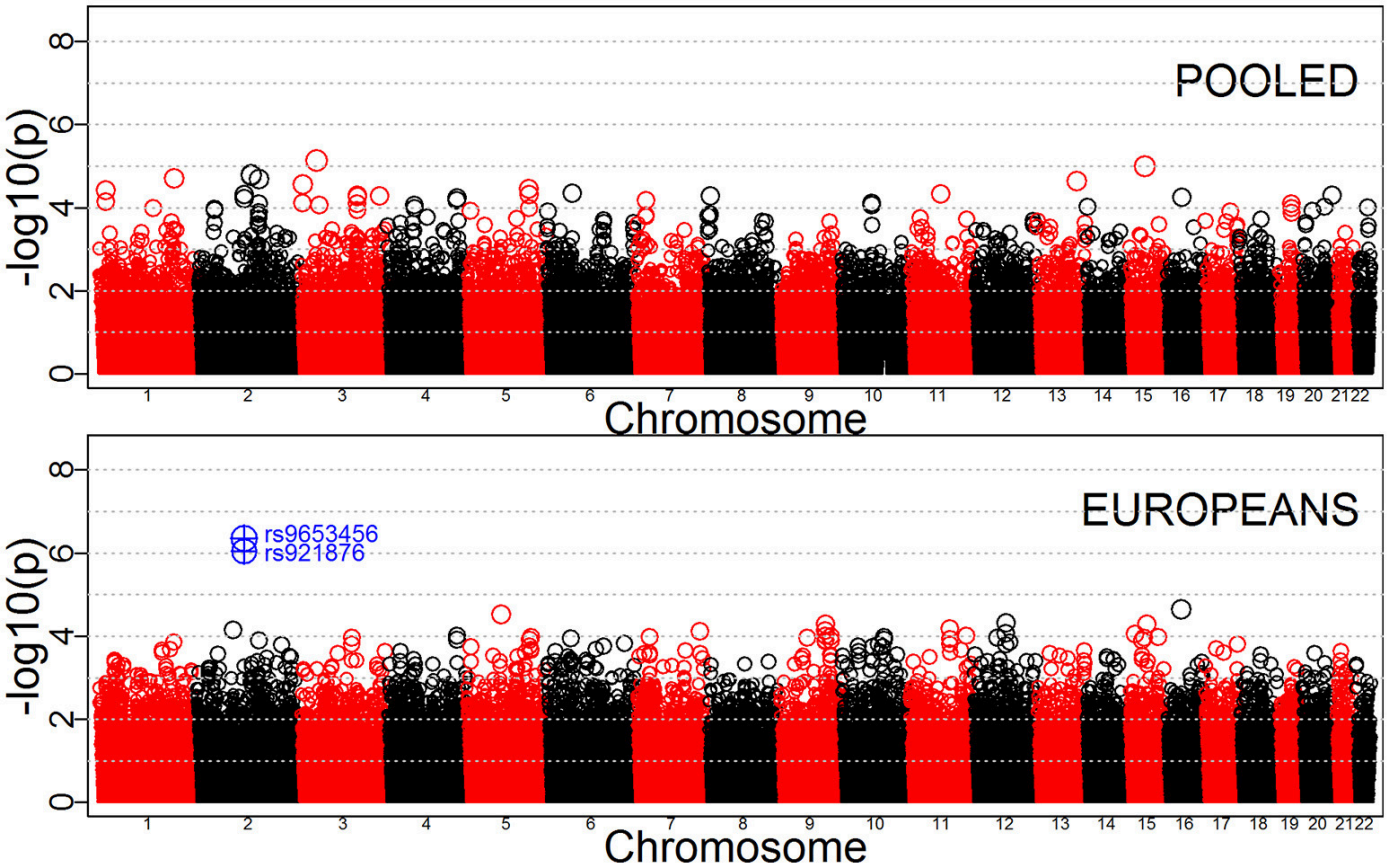
Manhattan plots for GxAlcohol effects in the pooled and Europeans-only analyses. SNPs with *p*-values below 10^−6^ are shown in blue.

Tables 3–5 provide the relative risk ratios (RRRs) and 95% confidence intervals (CIs) for the top 20 SNPs in each set of analyses depicted in Figure 1. These SNPs were ranked by *p*-value, and their corresponding *q*-values are also provided in these tables. RRRs were calculated as previously described by Haaland et al. (2017). Figures 2–4 show the QQ-plots for each maternal exposure. The corresponding Manhattan plots are provided in Figures 5–7. For SNPs that are not associated with isolated CL/P, –log_10_ (*p*-values) in the QQ-plots are expected to fall along the straight diagonal line representing the null distribution, within the 95% confidence band (gray area). Conversely, –log_10_ (*p*-values) would fall above this line, outside the confidence band and in the upper right-hand corner of the plot. Several SNPs were significantly associated with isolated CL/P in the GxVitamin analyses of the pooled sample (Figure 2). In the other analyses, none of the SNPs were outside the confidence bands, suggesting that the distribution of *p*-values is as expected if there are no associations. Still, in each of the GxVitamin and GxAlcohol analyses of the European sample (Tables 3, 5), two SNPs had markedly lower *p*-values than expected (see also Figures 5, 7). However, none of these SNPs was located in or near genes with obvious links to orofacial clefts. In the remainder of this section, therefore, we focus mainly on the results of the GxVitamin analysis of the pooled sample.

**Table 3:** Relative risk ratio (RRR) and 95% confidence interval (CI) for the top 20 SNPs in the GxVitamin analyses.

**Analysis**	**Top 20 SNPs^a^**	**Chromosomal band location^b^**	**Location of SNP in or near a given gene(s)^b^**	***p*-value**	***q*-value**	**RRR (95% CI)**
Pooled	**rs1339221**	1q41	In *ESRRG*	5.9e-08	0.011	0.56 (0.45–0.69)
	**rs11117745**	1q41	In *ESRRG*	6.8e-08	0.011	0.57 (0.46–0.70)
	**rs13027140**	2p16	Between *FLJ30838* and *LOC101927285*	1.0e-06	0.117	1.70 (1.38–2.11)
	**rs4233974**	2p16	Between *FLJ30838* and *LOC101927285*	2.2e-06	0.15	1.67 (1.35–2.06)
	**rs1316471**	7p12	Nearest gene is *COBL*	2.2e-06	0.15	2.36 (1.66–3.38)
	rs16970288	19q13.1	Between pseudogene *EEF1A1P7^a^* and *LINC01531*	6.2e-06	0.337	1.69 (1.35–2.13)
	rs2099557	1q41	In *ESRRG*	7.0e-06	0.337	0.62 (0.50–0.76)
	rs13022580	2q21.1	Between pseudogenes *RPL19P4* and *TEKT4P3*	8.4e-06	0.337	1.60 (1.30–1.96)
	rs5762534	22q12.1	In *TTC28*	9.0e-06	0.337	1.90 (1.43–2.51)
	rs938868	2q21.1	Between pseudogenes *MTND5P29* and *RPL19P4*	1.02e-05	0.343	1.59 (1.29–1.95)
	rs10992247	9q22.2	Between pseudogenes *IL6RP1* and *OR7E31P*	1.19e-05	0.367	0.42 (0.28–0.62)
	rs130413	22q12.1	In *TTC28*	1.61e-05	0.453	1.81 (1.38–2.37)
	rs10072077	5q14	Near *LOC101929423*	1.81e-05	0.468	2.35 (1.59–3.48)
	rs949771	2q21.1	In pseudogene *MTND5P29*	1.94e-05	0.468	1.56 (1.27–1.92)
	rs6696232	1p22.2	Between *ZNF644* and *HFM1*	2.12e-05	0.477	0.60 (0.47–0.76)
	rs247405	16q23	Nearest gene is *HSBP1*	2.59e-05	0.5	0.48 (0.34–0.67)
	rs621188	11q23.3	In *DSCAML1*	2.63e-05	0.5	1.59 (1.28–1.97)
	rs6431784	2p25.3	Nearest gene is *SOX11*	2.66e-05	0.5	1.74 (1.34–2.26)
	rs2042743	18p11.2	In *LDLRAD4*	3.04e-05	0.539	1.55 (1.26–1.91)
	rs11676593	2q21.1	Between pseudogenes *RPL19P4* and *TEKT4P3*	3.18e-05	0.539	1.56 (1.26–1.92)
European	**rs17734557**	7p12	Nearest gene is *COBL*	5e-07	0.163	3.22 (2.04–5.09)
	**rs1316471**	7p12	Nearest gene is *COBL*	1e-06	0.168	3.36 (2.07–5.46)
	rs10875883	12q13.1	Nearest gene is *CCDC65*	1.17e-05	0.607	0.49 (0.36–0.68)
	rs2838965	21q22.3	Nearest gene is *SLC19A1*	1.29e-05	0.607	0.49 (0.35–0.67)
	rs7741153	6p23	Nearest gene is *JARID2*	1.5e-05	0.607	0.45 (0.31–0.64)
	rs247405	16q23	Nearest gene is *HSBP1*	1.52e-05	0.607	0.23 (0.12–0.45)
	rs9544295	13q22	Nearest gene is *KCTD12*	1.63e-05	0.607	2.77 (1.74–4.41)
	rs13027140	2p16	Between *FLJ30838* and *LOC101927285*	1.83e-05	0.607	2.02 (1.46–2.78)
	rs2159963	12p13.3	Between *A2ML1* and *PHC1*	1.9e-05	0.607	2.35 (1.59–3.48)
	rs10746803	9q21.3	Between *C9orf170* and *DAPK1*	2.23e-05	0.607	0.34 (0.21–0.56)
	rs2575625	4q25	Between *OSTC* and *ETNPPL*	2.35e-05	0.607	1.96 (1.44–2.68)
	rs3792390	3q21	In *PDIA5*	2.46e-05	0.607	0.44 (0.30–0.65)
	rs17651808	15q21.2	Between *ARPP19* and *FAM214A*	2.49e-05	0.607	0.28 (0.15–0.50)
	rs4932844	19p12	In pseudogene *ZNF724*	2.53e-05	0.607	0.51 (0.37–0.70)
	rs295367	19p12	In pseudogene *LOC100132815*	3.21e-05	0.719	0.51 (0.38–0.70)
	rs9821623	3p22	In *ULK4*	3.66e-05	0.768	2.19 (1.51–3.18)
	rs2362803	19p12	Nearest gene is *ZNF724P*	4.16e-05	0.783	0.52 (0.38–0.71)
	rs260818	11q22.3	In *PDGFD*	4.2e-05	0.783	1.94 (1.41–2.67)
	rs10743549	12p13.3	Between *A2ML1* and *PHC1*	4.45e-05	0.786	2.33 (1.55–3.51)
	rs642307	1p32.1	In *C1orf87*	6.4e-05	0.905	1.89 (1.38–2.59)
Asian	rs13261120	8p22	In *SGCZ*	3.4e-06	0.73	2.45 (1.68–3.57)
	rs8055365	16q24	Between *TLDC1* and *COTL1*	6.2e-06	0.73	0.39 (0.26–0.59)
	rs12546303	8p22	In *SGCZ*	8.4e-06	0.73	2.35 (1.62–3.43)
	rs4773818	13q32	In *LOC101927284*	9.9e-06	0.73	3.16 (1.90–5.25)
	rs247832	16q24	Between *TLDC1* and *COTL1*	1.29e-05	0.73	0.42 (0.29–0.62)
	rs792306	13q32	In *LOC101927284*	1.33e-05	0.73	3.10 (1.86–5.17)
	rs12374531	5q32	Between *PPP2R2B* and *STK32A*	1.53e-05	0.73	3.22 (1.90–5.47)
	rs4695853	4q34	Nearest gene is *HAND2-AS1*	2.34e-05	0.897	3.12 (1.84–5.29)
	rs6860289	5p15.3	Nearest gene is *MTRR*	2.65e-05	0.897	2.35 (1.58–3.50)
	rs1615355	12q24.33	In *LOC100190940*	2.98e-05	0.897	2.29 (1.55–3.38)
	rs13418410	2p22	Between *RASGRP3* and *FAM98A*	3.24e-05	0.897	2.35 (1.57–3.52)
	rs423909	1p36.2	Nearest gene is *SLC45A1*	3.52e-05	0.897	2.18 (1.51–3.16)
	rs4670209	2p22	Between *RASGRP3* and *FAM98A*	3.59e-05	0.897	2.25 (1.53–3.31)
	rs1448561	2p22	Nearest gene is *FAM98A*	4.12e-05	0.897	2.32 (1.55–3.46)
	rs9286945	1p35	Nearest gene is *PTPRU*	4.49e-05	0.897	0.45 (0.30–0.66)
	rs2002022	13q21.3	Between pseudogenes *NPM1P22* and *OR7E111P*	4.6e-05	0.897	2.35 (1.56–3.54)
	rs17138910	10p13	In *RSU1*	5.05e-05	0.897	2.22 (1.51–3.26)
	rs4290780	3q13.2	In *CCDC80*	5.19e-05	0.897	3.85 (2.01–7.41)
	rs10503525	8p22	In *SGCZ*	5.96e-05	0.897	2.39 (1.56–3.65)
	rs13388224	2p22	Between *RASGRP3* and *FAM98A*	6.04e-05	0.897	2.27 (1.52–3.39)

a *SNPs with q-values <0.2 are shown in bold*.

b *Location of SNP was determined using the 1,000 Genomes browser at https://www.ncbi.nlm.nih.gov/variation/tools/1000genomes/*.

**Table 4:** Relative risk ratio (RRR) and 95% confidence interval (CI) for the top 20 SNPs in the GxSmoke analyses.

**Analysis^a^**	**Top 20 SNPs**	**Chromosomal band location^b^**	**Location of SNP in or near a given gene(s)^b^**	***p*-value**	***q*-value**	**RRR (95% CI)**
Pooled	rs820930	7q31.1	Nearest locus is *C7orf66*	1.06e-05	0.994	1.93 (1.44–2.58)
	rs210625	6q22.2	In *DCBLD1*	1.36e-05	0.994	1.79 (1.38–2.33)
	rs7258736	19q12	In *LOC101927210*	1.56e-05	0.994	2.99 (1.82–4.91)
	rs7247342	19q12	In *LOC101927210*	2.59e-05	0.994	2.89 (1.76–4.73)
	rs2024140	19q12	In *LOC101927210*	2.91e-05	0.994	2.97 (1.78–4.94)
	rs6810129	3q26.1	Nearest gene is *CT64*	2.94e-05	0.994	0.57 (0.44–0.74)
	rs1182865	6p21.2	Between *KCNK5* and *KCNK17*	3.43e-05	0.994	0.51 (0.37–0.70)
	rs1013592	19q12	In *LOC101927210*	3.47e-05	0.994	2.81 (1.72–4.57)
	rs10085496	7p21	Between pseudogenes *GAPDHP68* and *PER4*	3.85e-05	0.994	0.58 (0.45–0.75)
	rs4805661	19q12	Nearest gene is *TSHZ3*	4.05e-05	0.994	0.57 (0.43–0.74)
	rs2543146	8p22	In *TUSC3*	4.19e-05	0.994	1.73 (1.33–2.24)
	rs4804843	19q12	In *LOC101927210*	4.41e-05	0.994	2.86 (1.73–4.73)
	rs10417454	19q12	In *LOC101927210*	4.63e-05	0.994	2.84 (1.72–4.69)
	rs244226	21q21	Between pseudogenes *MAPK6PS2* and *ZNF299P*	4.88e-05	0.994	1.72 (1.32–2.23)
	rs352806	8p22	In *TUSC3*	5.21e-05	0.994	0.53 (0.38–0.72)
	rs2191018	19q12	In *LOC101927210*	5.4e-05	0.994	2.82 (1.71–4.67)
	rs6806286	3q22	In *CPNE4*	6.37e-05	0.994	1.72 (1.32–2.25)
	rs2834061	21q22.1	Nearest gene is *OLIG2*	6.37e-05	0.994	1.80 (1.35–2.41)
	rs12204228	6q22.1	Between *RFX6* and *VGLL2*	6.44e-05	0.994	0.40 (0.25–0.63)
	rs11014205	10p12.2	In *ARHGAP21*	8.06e-05	0.994	0.55 (0.41–0.74)
European	rs10106898	8q22.1	Between pseudogenes *GAPDHP30* and *TUBBP7*	4.1e-06	0.684	0.29 (0.18–0.50)
	rs10095562	8q22.1	Between pseudogenes *GAPDHP30* and *TUBBP7*	1.4e-05	0.684	0.30 (0.17–0.52)
	rs10282921	8q22.1	Between pseudogenes *GAPDHP30* and *TUBBP7*	1.43e-05	0.684	0.30 (0.18–0.52)
	rs820930	7q31.1	Between *LOC646614* and *LOC100421901*	1.45e-05	0.684	2.11 (1.51–2.97)
	rs266642	5q23.2	Nearest gene is *GRAMD3*	1.55e-05	0.684	2.69 (1.72–4.22)
	rs601904	11q13.4	Between peudogene *CYCSP27* and *LIPT2*	1.59e-05	0.684	0.49 (0.35–0.68)
	rs17608059	17p12	Nearest gene is *COX10-AS1*	1.68e-05	0.684	1.97 (1.44–2.67)
	rs657339	11q13.4	In *LOC100287896*	1.85e-05	0.684	0.49 (0.35–0.68)
	rs617989	11q13.4	In *LIPT2*	1.9e-05	0.684	0.49 (0.36–0.68)
	rs649460	11q13.4	Nearest gene is *LIPT2*	2.28e-05	0.684	0.49 (0.36–0.68)
	rs2509563	11q13.4	Nearest gene is *LIPT2*	2.29e-05	0.684	0.50 (0.36–0.69)
	rs1013592	19q12	In *LOC101927210*	2.45e-05	0.684	3.84 (2.05–7.17)
	rs1783196	11q13.4	Nearest gene is *LIPT2*	2.85e-05	0.684	0.50 (0.36–0.69)
	rs648677	11q13.4	Between *LIPT2 and POLD3*	2.85e-05	0.684	0.50 (0.36–0.69)
	rs10028174	4q22	In *CCSER1*	3.65e-05	0.796	0.40 (0.26–0.62)
	rs1655475	11q13.4	Between *LIPT2 and POLD3*	3.79e-05	0.796	0.51 (0.37–0.70)
	rs2089253	4q21.1	In *USO1*	4.34e-05	0.858	0.28 (0.16–0.52)
	rs3317	5q22	In *REEP5* and *SRP19*	5.55e-05	0.89	0.53 (0.39–0.72)
	rs11692230	2q12	In *IL1RL2*	5.67e-05	0.89	1.89 (1.39–2.58)
	rs4805661	19q12	Nearest gene is *TSHZ3*	6.19e-05	0.89	0.53 (0.38–0.72)

a *We were unable to assess GxSmoke effects in the Asian sample because very few of the mothers reported smoking cigarettes in the periconceptional period*.

b *Location of SNP was determined using the 1,000 Genomes browser at https://www.ncbi.nlm.nih.gov/variation/tools/1000genomes/*.

**Table 5:** Relative risk ratio (RRR) and 95% confidence interval (CI) for the top 20 SNPs in the GxAlcohol analyses.

**Analysis^a^**	**Top 20 SNPs^b^**	**Chromosomal band location^c^**	**Location of SNP in or near a given gene(s)^c^**	***p*-value**	***q*-value**	**RRR (95% CI)**
Pooled	rs17023089	3p24.1	Nearest gene is *RBMS3*	7.4e-06	0.858	0.32 (0.19–0.53)
	rs7164773	15q22.2	In *RORA*	9.8e-06	0.858	1.73 (1.35–2.20)
	rs6748903	2q14.3	Near *LOC101927881*	1.65e-05	0.858	0.51 (0.38–0.69)
	rs11691558	2q21.1	In *LOC101927881*	1.72e-05	0.858	0.51 (0.38–0.69)
	rs4132008	1q32.1	In *PLEKHA6*	1.94e-05	0.858	1.75 (1.35–2.27)
	rs2118769	2q22.3	Nearest gene is *ACVR2A*	2.04e-05	0.858	0.58 (0.46–0.75)
	rs1335594	13q33	In *ITGBL1*	2.3e-05	0.858	1.74 (1.35–2.25)
	rs3805025	3p26	In *ITPR1*	2.77e-05	0.858	1.96 (1.43–2.69)
	rs30306	5q32	Nearest gene is *ADBR2*	3.58e-05	0.858	1.66 (1.30–2.11)
	rs6685648	1p36.2	In *CASP9*	3.89e-05	0.858	0.59 (0.46–0.76)
	rs2064317	6p21.3	In *TULP1*	4.45e-05	0.858	1.65 (1.30–2.11)
	rs686805	11q13.4	In *SHANK2*	4.66e-05	0.858	2.24 (1.52–3.30)
	rs9653456	2q12	Nearest gene is *EDAR*	4.76e-05	0.858	1.85 (1.37–2.49)
	rs2163752	5q32	Between *HTR4* and *ADRB2*	4.77e-05	0.858	1.64 (1.29–2.09)
	rs13086826	3q22	In *CPNE4*	4.94e-05	0.858	0.59 (0.45–0.76)
	rs4810165	20q13.3	Between *EDN3* and peudogene *PIEZO1P1*	5.01e-05	0.858	1.75 (1.34–2.30)
	rs4859190	3q27	Nearest gene is *MCF2L2*	5.12e-05	0.858	0.58 (0.45–0.76)
	rs2740882	8p23.3	In *CSMD1*	5.15e-05	0.858	0.48 (0.33–0.68)
	rs13077768	3q22	In *CPNE4*	5.29e-05	0.858	0.59 (0.45–0.76)
	rs11076508	16q12.1	Between *ZNF423* and pseudogene *RPL34P29*	5.56e-05	0.858	0.61 (0.48–0.78)
European	**rs9653456**	2q12	Between *EDAR* and *SH3RF3*	4e-07	0.148	2.40 (1.71–3.37)
	**rs921876**	2q12	Nearest gene is *EDAR*	9e-07	0.153	2.09 (1.56–2.81)
	rs11076508	16q12.1	Between *ZNF423* and pseudogene *RPL34P29*	2.25e-05	0.892	0.54 (0.40–0.72)
	rs7713774	5q14	In *ACOT12*	3.05e-05	0.892	0.51 (0.37–0.70)
	rs10784765	12q15	Between *CPM* and *CPSF6*	4.76e-05	0.892	0.41 (0.27–0.63)
	rs893912	15q22.3	Between *LCTL* and *SMAD6*	5.04e-05	0.892	2.28 (1.53–3.39)
	rs17199679	9q31	Between pseudogene *RPL36AP35* and *ACTL7B*	5.1e-05	0.892	2.86 (1.72–4.76)
	rs3020054	11q21	Nearest gene is *CCDC67*	6.7e-05	0.892	0.52 (0.38–0.72)
	rs2192926	2p12	Nearest gene is *TACR1*	7.24e-05	0.892	0.50 (0.36–0.71)
	rs7862797	9q31	Between pseudogene *RPL36AP35* and *ACTL7B*	7.49e-05	0.892	2.77 (1.67–4.58)
	rs851674	7q35	In *CNTNAP2*	7.85e-05	0.892	1.87 (1.37–2.56)
	rs6581864	12q15	Between *CPM* and *CPSF6*	8.72e-05	0.892	1.81 (1.34–2.43)
	rs6495940	15q14	Between *TMCO5A* and *SPRED1*	8.82e-05	0.892	0.41 (0.26–0.64)
	rs6822683	4q34	Nearest gene is *GALNT7*	9.58e-05	0.892	1.82 (1.35–2.46)
	rs2276049	11q24	In *VWA5A*	9.78e-05	0.892	0.48 (0.33–0.69)
	rs2417976	9q31	Between *TMEM245* and *FRRS1L*	9.82e-05	0.892	2.04 (1.42–2.91)
	rs10759878	9q33	In *ASTN2*	9.89e-05	0.892	1.93 (1.39–2.69)
	rs4778036	15q26.1	Between *SLCO3A1* and pseudogene *DUXAP6*	0.0001033	0.892	1.80 (1.34–2.43)
	rs17104078	10q23.1	Nearest gene is *CCSER2*	0.0001042	0.892	2.48 (1.57–3.93)
	rs12540601	7p15.3	Between *TRA2A* and pseudogene *LOC442517*	0.0001044	0.892	0.56 (0.42–0.75)

a *We were unable to assess GxAlcohol effects in the Asian sample because very few of the mothers reported drinking alcohol in the periconceptional period*.

b *SNPs with q-values <0.2 are shown in bold*.

c *Location of SNP was determined using the 1,000 Genomes browser at https://www.ncbi.nlm.nih.gov/variation/tools/1000genomes/*.

**Figure 8:**
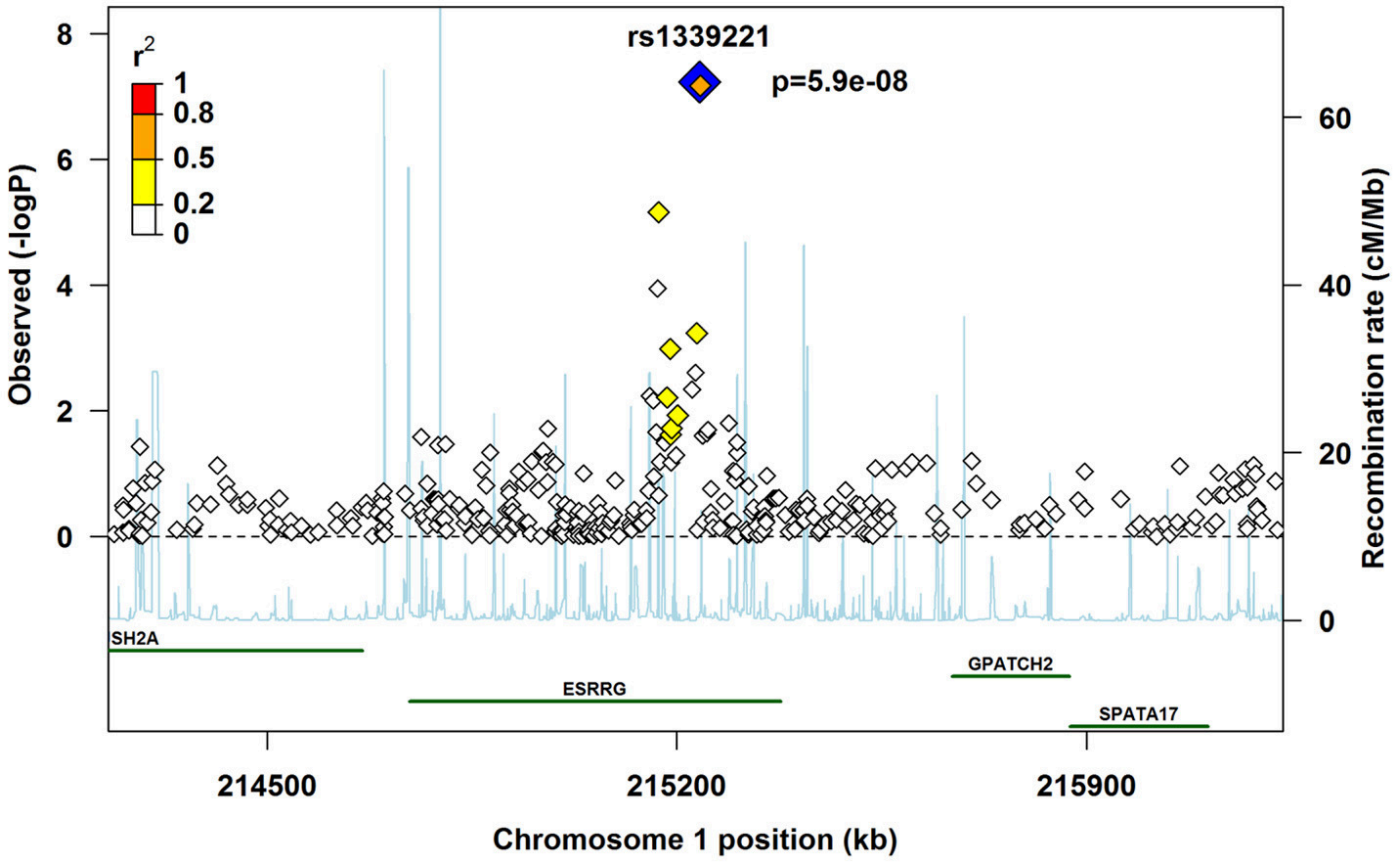
Regional association plot to assess the strength of association and regional information flanking the lead SNP in *ESRRG*, rs1339221, shown here in blue alongside its *p*-value.

All the top 20 SNPs in the GxVitamin analysis of the pooled sample had *q*-values below 0.54 (Table 3). The two top SNPs among these, rs1339221 and rs11117745, both had a *q*-value of 0.01 and are located in the gene for “Estrogen-related receptor gamma” (*ESRRG*). These SNPs are in strong linkage disequilibrium (LD) with one another (*R*^2^ = 0.77 and D' = 1.00). A third SNP, rs2099557 (*q* = 0.037), is also located in *ESRRG* but is in weaker LD with the above two SNPs (*R*^2^ = 0.33 and D' = 0.57 with rs1339221; *R*^2^ = 0.28 and D' = 0.61 with rs11117745). A regional plot centered around the lead SNP in *ESRRG* (rs1339221) was generated to visualize the strength of the association signals and regional information around that SNP (Figure 8). Next, we performed stratified analyses of the SNPs in *ESRRG* by first testing for an overall child effect in the unstratified sample, followed by the effects among children who were exposed and unexposed to maternal vitamin use, respectively (Table 6). All three SNPs exhibited a so-called “qualitative interaction” (Clayton, 2009), in that the effect of the SNP-allele among mothers taking vitamins was in the opposite direction of that among mothers not taking vitamins. Specifically, RR>1 among non-takers, whereas RR < 1 among takers, and none of the 95% CIs included 1 (Table 6). There were no statistically significant overall effects of the child's allele alone for any of the SNPs; all the 95% CIs included RR = 1. As mentioned earlier, the total GxE effect was measured as the ratio of the RRs in each stratum of vitamin use (i.e., RR_Vitamins_/RR_No_ _vitamins_). RRRs and 95% CIs were 0.56 (0.45–0.69) with the variant allele at rs1339221 (*q* = 0.011), 0.57 (0.46–0.70) with the variant allele at rs11117745 (*q* = 0.011), and 0.62 (0.50–0.76) with the variant allele at rs2099557 (*q* = 0.037) (Table 6).

**Table 6:** Stratified analyses of the top three SNPs in *ESRRG*.

**SNP name^a^**	**Stratum**	**RR (95% CI)^b^**	***p*-value**	**Frequency**
rs1339221	All (child effect)	0.94 (0.84–1.04)	0.22	0.40
	No vitamin use	1.17 (1.03–1.33)	0.016	0.38
	Vitamin use	0.66 (0.56–0.78)	5 × 10^−7^	0.43
	Across strata	0.56 (0.45–0.69)	6 × 10^−8^	–
rs11117745	All (child effect)	0.96 (0.87–1.06)	0.41	0.46
	No vitamin use	1.19 (1.05–1.35)	0.0064	0.46
	Vitamin use	0.68 (0.58–0.80)	2 × 10^−6^	0.46
	Across strata	0.57 (0.46–0.70)	7 × 10^−8^	–
rs2099557	All (child effect)	0.96 (0.87–1.06)	0.40	0.40
	No vitamin use	1.15 (1.01–1.31)	0.031	0.37
	Vitamin use	0.71 (0.61–0.84)	5 × 10^−5^	0.44
	Across strata	0.62 (0.50–0.76)	5 × 10^−6^	–

a *All three SNPs are located within chromosomal region 1q41 according to the 1000 Genomes browser (https://www.ncbi.nlm.nih.gov/variation/tools/1000genomes/)*.

b *Relative risk (RR) for the overall child effect, and effects within each stratum of vitamin use. Across strata, an effect is measured as the ratio of the RRs (i.e., RRR) for vitamin use and no vitamin use*.

Next, we considered haplotypes of the top three SNPs in *ESRRG* (Table 7) and analyzed the following two-SNP and three-SNP combinations: (i) rs2099557-rs1339221, (ii) rs1339221-rs11117745, and (iii) rs2099557-rs1339221-rs11117745. The RRRs and 95% CIs were 0.52 (0.41-0.66) for SNP combination (i), 0.55 (0.44–0.68) for SNP combination (ii), and 0.50 (0.40–0.64) for SNP combination (iii) (Table 7). The pattern of RRRs was similar to that in the single-SNP analyses. Provided that vitamin use itself is not harmful, the haplotypes with the lowest *p*-values appeared to be protective among vitamin-takers, but detrimental among non-takers (Table 7). The RRs and RRRs were even further away from 1, and the *p*-values were lower than in the single-SNP analyses. The most prominent effect was with the “t-t-t” haplotype in the analysis of SNP combination (iii) above, suggesting that the observed effects with SNP combinations (i) and (ii), as well as those observed in the single-SNP analyses, are likely to be driven by this haplotype.

**Table 7:** Haplotype analysis for the top three SNPs in *ESRRG*.

**SNP combination^a^**	**Haplotype**	**Top^b^**	**Ref.^c^**	**Frequency (top vs. ref)**	**Stratum**	**RR (95% CI)^d^**	***p*-value**
rs2099557-rs1339221	t/C × t/C	t-t	C-C	0.30 vs. 0.50	All (child effect)	0.93 (0.83–1.04)	0.20
				0.27 vs. 0.51	No vitamin use	1.21 (1.04–1.40)	0.013
				0.35 vs. 0.48	Vitamin use	0.63 (0.53–0.75)	6 × 10^−7^
				–	Across strata	0.52 (0.41–0.66)	6 × 10^−8^
rs1339221-rs11117745	t/C × t/G	t-t	C-G	0.40 vs. 0.54	All (child effect)	0.94 (0.85–1.04)	0.25
				0.39 vs. 0.54	No vitamin use	1.19 (1.04–1.36)	0.0091
				0.43 vs. 0.54	Vitamin use	0.65 (0.55–0.77)	6 × 10^−7^
				–	Across strata	0.55 (0.44–0.68)	4 × 10^−8^
rs2099557-rs1339221-rs11117745	t/C × t/C × t/G	t-t-t	C-C-G	0.30 vs. 0.46	All (child effect)	0.94 (0.83–1.05)	0.27
				0.27 vs. 0.45	No vitamin use	1.24 (1.07–1.45)	0.0061
				0.35 vs. 0.46	Vitamin use	0.63 (0.52–0.75)	7 × 10^−7^
				–	Across strata	0.50 (0.40–0.64)	2 × 10^−8^

a *All three SNPs are located within the chromosomal band 1q41 according to the 1,000 Genomes browser (https://www.ncbi.nlm.nih.gov/variation/tools/1000genomes/)*.

b *“Top” refers to the haplotype with the lowest p-value in the analysis*.

c *The most frequent haplotype was used as reference*.

d *Relative risk (RR) for the overall child effect, and effects within each stratum of vitamin use. Across strata, an effect is measured as the ratio of the RRs (i.e., RRR) for vitamin use and no vitamin use*.

**Figure 9:**
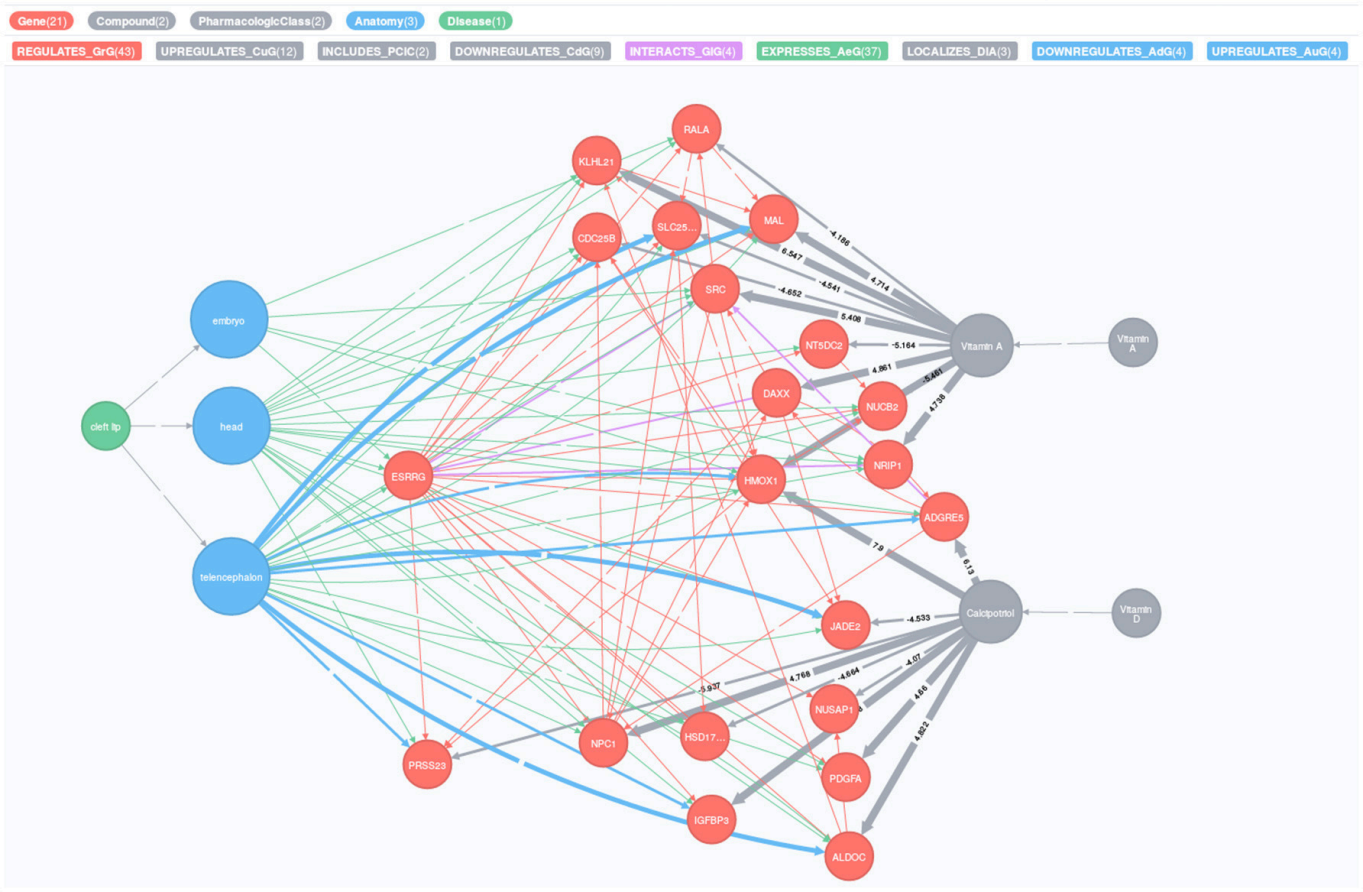
Relationships between cleft lip, vitamins and *ESRRG*, visualized as a heterogenic network, where each relationship evidenced in public databases is depicted as an arrow connecting two nodes. Different types of nodes (e.g., genes, diseases, etc.) are connected by different types of relationships (e.g., regulation, interaction, etc.). The nodes and arrows are colored according to the legend at the top of the figure. The thickness of the “DOWNREGULATES_AdG” arrow is less than that of “UPREGULATES_AuG,” and similarly for “DOWNREGULATES_CdG” and “UPREGULATES_CuG.” The numbers on the two latter arrows show the Z-score, which is a measure for the strength of the influence. The figure was generated using the Hetionet network (see the Materials and Methods section for details).

Our *in silico* analyses using Hetionet revealed that the cleft lip phenotype is influenced by vitamins A and D through a network of genes connected to *ESRRG* (Figure 9). Except for “Platelet derived growth factor subunit A” (*PDGFA*), few of the genes in the network have previously been linked to orofacial clefts. Figure 9 shows that *ESRRG* is expressed in three major tissues/organs and that the cleft lip phenotype is localized to the embryo, head, and telencephalon (the most highly developed and anterior part of the forebrain). *ESRRG* regulates or interacts with various genes that are regulated by the levels of vitamin A and D. Note that several genes are up- or down-regulated in the telencephalon (marked by blue arrows in Figure 9) and they also interact directly with *ESRRG*.

## Discussion

The current genome-wide search for GxE effects in isolated CL/P is based on the largest available GWAS dataset on orofacial cleft triads (Beaty et al., 2010). We identified a statistically significant GxE effect between maternal periconceptional vitamin use and genetic variants in *ESRRG* in the pooled analysis. We also identified potential GxVitamin and GxAlcohol effects in the European sample (Tables 3, 5), but none of the SNPs were outside the 95% confidence bands in the QQ plots (Figures 2, 4), nor were they located in or near genes with obvious connections to orofacial clefts. Because of a lack of observations, we were unable to perform GxSmoke and GxAlcohol analyses in the Asian sample. The low level of alcohol intake and cigarette smoking appears to be a general trend among Asian women, and is likely to be even lower among those who are pregnant or planning pregnancy (Yang et al., 2010; Ng et al., 2014; Haaland et al., 2017). Although GxVitamin analysis of the Asian sample was not hampered by a lack of observations, vitamin intake was nevertheless markedly lower in this group compared to European women (14 vs. 56%) (Figure 1).

The current genome-wide scan for GxE effects in isolated CL/P was motivated by a number of observations. First, several studies have reported associations between orofacial clefts and periconceptional maternal cigarette smoking, alcohol intake and vitamin use (reviewed in Jugessur et al., 2009a; Dixon et al., 2011; Marazita, 2012; Rahimov et al., 2012; Leslie and Marazita, 2013; Beaty et al., 2016), but our comprehensive analysis of 334 autosomal cleft candidate genes showed little evidence of interaction with these maternal exposures despite being the largest GxE study of clefts at the time (Skare et al., 2012). Second, the lack of GxE effects may be due to a combination of limited statistical power to detect nothing but the largest GxE effects and the *a priori* small environmental contributions to CL/P (~9%) and CPO (~10%) (Grosen et al., 2011). Because most previous GxE studies have used a candidate-gene approach and are based on relatively small sample sizes, the small environmental contributions are likely to have further reduced the power to detect a GxE effect. Third, only two genome-wide studies of GxE effects have so far been published in orofacial clefts—in isolated CPO (Beaty et al., 2011; Wu et al., 2014). We thus focused on the larger sample of isolated CL/P and screened for GxE effects using the same GWAS dataset as in Wu et al. (2014).

The connection between vitamins and variants in *ESRRG* is novel in the context of orofacial clefts. To shed more light on how vitamins, *ESRRG* and clefting might relate to one another, we performed *in silico* analyses using the Hetionet database (Figure 9). By harnessing data from several publicly available meta-databases, Hetionet generates a detailed overview of the relationships between a given disorder/disease, genes and compounds that might easily be overlooked if the focus were solely on specific aspects of disease-gene associations (Greene et al., 2015; Himmelstein et al., 2017). Our analyses revealed a rich network of genes connecting cleft lip to *ESRRG* and to two vitamins in particular—vitamins A and D. These genes are significantly influenced by the levels of these two vitamins, which might partially explain why the extra vitamin intake by pregnant mothers appears to protect the fetus against clefts. There is some evidence in the literature linking vitamin A itself and genes related to vitamin A, e.g., retinoic acid receptor alpha, *RARA*, with the risk of orofacial clefts (Rothman et al., 1995; Mitchell et al., 2003; Bille et al., 2007; Johansen et al., 2008; Boyles et al., 2009; Skare et al., 2012). For instance, early studies in mice showed that the timing of exposure to retinoic acid (a metabolite of vitamin A) is important in the disruption of the expression patterns of key growth factors, resulting in abnormally small palatal shelves that cannot fuse (Abbott et al., 1989, 2005; Abbott and Birnbaum, 1990). Compared to vitamin A or vitamin B complex, few studies have examined the effects of high or low dose of vitamin D on clefting risk. Unfortunately, the cleft consortium (Beaty et al., 2010) did not include detailed information on the use of different types of vitamins, which would have allowed a more targeted analysis of vitamins A and D in relation to the *ESRRG* variants.

The impact of specific variants in *ESRRG* on the risk of orofacial clefts is also uncharted territory, but several lines of evidence point to a biologically plausible link between estrogens, *ESRRG* and craniofacial malformations. First, estrogens are a group of steroid-based sex hormones that are involved in several important developmental and physiological processes, including cartilage proliferation and growth, and formation of the craniofacial complex (Ahi, 2016). Second, sex hormones are involved in several traits associated with sexual dimorphism (Callewaert et al., 2010; Randall et al., 2013; Sanger et al., 2014). Given the consistently observed higher male-to-female ratio of isolated CL/P (~2:1 in Caucasians), it is plausible that the skewed sex prevalence is a manifestation of opposing sex steroid actions. Third, exposure to very high or very low doses of estrogens during embryonic development results in craniofacial skeletal malformations in various animal models (Fushimi et al., 2009; Cohen et al., 2014; Morthorst et al., 2016). For instance, when zebrafish are exposed to bisphenol-A (an endocrine-disrupting chemical that mimics estrogen), they develop craniofacial malformations (Kramer et al., 1990). Signaling and dosage regulation of estrogens are finely orchestrated by estrogen receptors (ERs) and estrogen related receptors (ESRRs). These two closely-related families of receptors share target genes, co-regulators and promoters (Maglott et al., 2005). *ESRRG*, the gene identified in our GxVitamin analyses, encodes the orphan nuclear receptor “Estrogen-related receptor γ” (ERRγ). ERRγ itself does not appear to be important for skeletal development, but it is a sex-dependent negative regulator of postnatal bone formation (Cardelli and Aubin, 2014).

The majority of the genes in Figure 9 have not previously been linked to orofacial clefts, except perhaps for *PDGFA*. This gene belongs to the PDGF family of genes that play important roles in the PDGF receptor-alpha (PDGFR-α) signaling pathway. Compared to *PDGFA, PDGFC* has a well-substantiated role in palatogenesis (Eberhart et al., 2008). Mice with the *Pdgfc* gene knocked out (*Pdgfc*^−/−^) exhibit a complete cleft of the secondary palate (Ding et al., 2004). The phenotype is less severe in *Pdgfa*^−/−^ mice. The ligands PDGFA and PDGFC share a common pathway with the PDGFR-α receptor in regulating the development of craniofacial structures. Targeted deletion of murine *Pdgfra* causes neural tube closure defects, including midfacial and palatal clefts (Soriano, 1997). Retinoic acid, on the other hand, inhibits proliferation of embryonic palatal shelves in mice by downregulating *Pdgfc* activity (Han et al., 2006). Pedigo et al. (2007) later reported the location of a complex retinoic acid response element in a region upstream of the transcription start site of *PDGFA* that was previously shown to harbor basal and vitamin D-inducible enhancer activity, which lends support to the connections seen between these entities in Figure 9.

**Figure 10:**
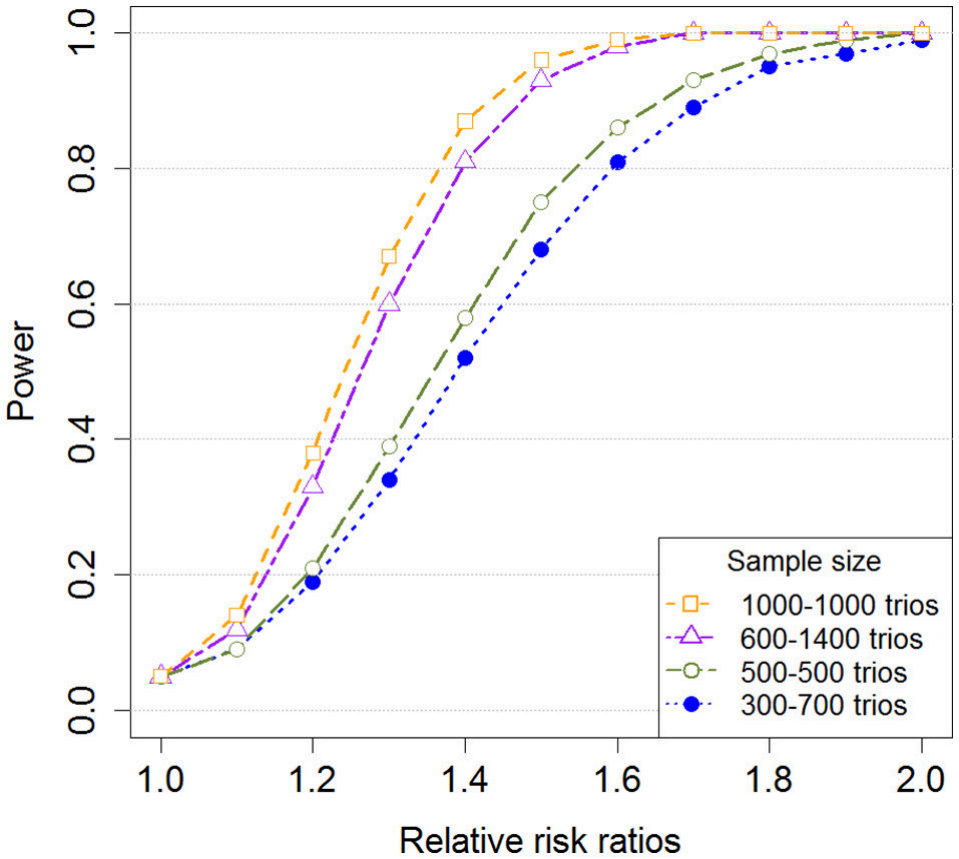
Single-SNP power for different sample sizes and proportions of exposed to unexposed mothers. The figure shows the power for detecting a GxE effect with increasing relative risk ratios (RRR). The relative risk in the smallest exposure group was increased, but the relative risk in the largest exposure group was set equal to 1. Additionally, we used a minor allele frequency (MAF) of 0.2, a nominal significance level of 5%, and a total sample size of 1,000 triads and 2,000 triads with different proportions of exposed/unexposed mothers. Note that the minor allele was used as the risk allele.

In addition to the novel insights from the biological findings, we aimed at developing a robust method for genome-wide screening of GxE effects in GWAS data. Using a log-linear model with multiplicative dose-response is a very efficient statistical approach, and case-parent triads provide sufficient information to reconstruct haplotypes with high precision. Yet, our study may still lack power to detect effects that are small. Figure 10 depicts power simulations for different sample sizes and different proportions of exposed to unexposed mothers, reflecting those that were available in the current GWAS dataset. The figure shows that there is acceptable power at a nominal significance level of 5% to detect a single-SNP GxE effect with relative risk ratio (RRR) of 1.4 or higher in a sample consisting of 2,000 triads, and an effect with RRR of 1.6 or higher with 1,000 triads. The proportion of exposed to unexposed cases does not have a major impact on power as long as it does not deviate substantially from 1-to-1. In a GWAS setting, there is of course the extra burden of extensive multiple testing. Controlling the FDR in our study for each exposure is less taxing on statistical power and better suited for discovery than, for instance, a strict Bonferroni correction. However, it increases the need for independent verification of top hits.

Our study has limitations. Even though it was based on the largest collection of isolated CL/P triads to date, it still had limited statistical power in the smallest group of exposed mothers. This was especially true for the GxSmoke and GxAlcohol analyses in the Asian sample, where the low level of exposure prevented any meaningful GxE analysis. The lack of a replication cohort was also a major shortcoming. Our study is the first to investigate the risk of isolated CL/P using GWAS data in two major ethnicities, and there are currently no other similar studies on isolated CL/P that can be used to confirm our findings. Further, our assumption of a detectable RRR of 1.4 with a sample size of 2,000 triads and an RRR of 1.6 with 1,000 triads may be optimistic in the context of birth defects and complex traits in general. The current scan for GxE effects in isolated CL/P should therefore be considered exploratory and hypothesis-generating at this stage, assuming that other researchers who in the future have access to comparable cleft data and more detailed information on different types of vitamins (including vitamins A and D) would be interested in replicating these findings.

To summarize, we identified significant interactions between variants in *ESRRG* and vitamin use in the pooled analysis. Our *in silico* analyses revealed an intricate network of genes linking cleft lip, *ESRRG* and two vitamins in particular: vitamins A and D. These GxE effects are novel and warrant further investigations to unravel the potential interplay between vitamins and *ESRRG* variants. If confirmed in other cleft samples, they could provide prospects for exploring the impact of estrogens and vitamins on orofacial clefts, with potential translational applications.

## Author contributions

AJ, HG, RL, ØH, MG, and JR: Conception of the work, study design, data analysis and interpretation, manuscript preparation and final manuscript approval; HG, MG, JR, and ØH: Statistical modeling and software design; AJ, HG, and RL: Funding acquisition.

### Conflict of interest statement

The authors declare that the research was conducted in the absence of any commercial or financial relationships that could be construed as a potential conflict of interest.
