# Supplementary material for: A Genome-Wide Search for Gene-Environment Effects in Isolated Cleft Lip with or without Cleft Palate Triads Points to an Interaction between Maternal Periconceptional Vitamin Use and Variants in ESRRG
Source: Front Genet. 2018 Feb 26;9:60. doi: 10.3389/fgene.2018.00060 (PMC5834486; doi:10.3389/fgene.2018.00060)
Supplement: Supplementary file 1 [file Presentation1.PDF]

August 11, 2017
Original code used in Neo4j to query the Hetionet database (Himmelstein, D. S. et al. 1–46 (2016) doi:10.1101/087619)

```
Server version Neo4j/3.3.0-alpha05
Server address localhost:7687
Query
MATCH path = allShortestPaths((node1:Gene)-[*..3]-(node2:PharmacologicClass))
WHERE
  node1.name = 'ESRRG' AND node2.name STARTS WITH 'Vitamin'
WITH relationships(path) AS rels
UNWIND rels AS rel
RETURN
  DISTINCT startNode(rel) AS source, endNode(rel) AS target, rel AS relationship
UNION
MATCH path = allShortestPaths((node1:Gene)-[*..3]-(node2:Disease))
WHERE
  node1.name = 'ESRRG' AND node2.name = 'cleft lip'
WITH relationships(path) AS rels UNWIND rels AS rel
RETURN
  DISTINCT startNode(rel) AS source, endNode(rel) AS target, rel AS relationship
```

Response


```
[
  {
    "keys": [
      "source",
      "target",
      "relationship"
    ],
    "length": 3,
    "_fields": [
      {
        "identity": {
          "low": 31332,
          "high": 0
        },
        "labels": [
          "Gene"
        ],
        "properties": {
          "license": "CC0 1.0",
          "identifier": {
            "low": 2104,
            "high": 0
          },
          "chromosome": "1",
          "name": "ESRRG",
          "description": "estrogen-related receptor gamma",
          "source": "Entrez Gene",
          "url": "http://identifiers.org/ncbigene/2104"
        }
      },
      {
        "identity": {
          "low": 24096,
          "high": 0
        },
        "labels": [
          "Gene"
        ],
```

```
        "properties": {
          "license": "CC0 1.0",
          "identifier": {
            "low": 3162,
            "high": 0
          },
          "chromosome": "22",
          "name": "HMOX1",
          "description": "heme oxygenase 1",
          "source": "Entrez Gene",
          "url": "http://identifiers.org/ncbigene/3162"
        }
      },
      {
        "identity": {
          "low": 10859,
          "high": 0
        },
        "start": {
          "low": 31332,
          "high": 0
        },
        "end": {
          "low": 24096,
          "high": 0
        },
        "type": "REGULATES_GrG",
        "properties": {
          "method": "measured",
          "unbiased": true,
          "source": "LINCS L1000",
          "subtypes": [
            "overexpression downregulates"
          ]
        }
      }
    ],
    "_fieldLookup": {
      "source": 0,
      "target": 1,
      "relationship": 2
    }
  },
  {
    "keys": [
      "source",
      "target",
      "relationship"
    ],
    "length": 3,
    "_fields": [
      {
        "identity": {
          "low": 46428,
          "high": 0
        },
        "labels": [
          "Compound"
        ],
        "properties": {
          "license": "CC BY-NC 4.0",
```

```
          "identifier": "DB02300",
          "inchikey": "InChIKey=LWQQLNNNIPYSNX-UROSTWAQSA-N",
          "inchi": "InChI=1S/C27H40O3/c1-17(6-13-25(29)20-8-9-20)23-11-12-24-
          19(5-4-14-27(23,24)3)7-10-21-15-22(28)16-26(30)18(21)2/h6-7,10,13,
          17,20,22-26,28-30H,2,4-5,8-9,11-12,14-16H2,1,3H3/b13-6+,19-7+,21-
          10-/t17-,22-,23-,24+,25-,26+,27-/m1/s1",
          "name": "Calcipotriol",
          "source": "DrugBank",
          "url": "http://www.drugbank.ca/drugs/DB02300"
        }
      },
      {
        "identity": {
          "low": 24096,
          "high": 0
        },
        "labels": [
          "Gene"
        ],
        "properties": {
          "license": "CC0 1.0",
          "identifier": {
            "low": 3162,
            "high": 0
          },
          "chromosome": "22",
          "name": "HMOX1",
          "description": "heme oxygenase 1",
          "source": "Entrez Gene",
          "url": "http://identifiers.org/ncbigene/3162"
        }
      },
      {
        "identity": {
          "low": 815082,
          "high": 0
        },
        "start": {
          "low": 46428,
          "high": 0
        },
        "end": {
          "low": 24096,
          "high": 0
        },
        "type": "UPREGULATES_CuG",
        "properties": {
          "method": "measured",
          "unbiased": true,
          "z_score": 7.9,
          "source": "LINCS L1000"
        }
      }
    ],
    "_fieldLookup": {
      "source": 0,
      "target": 1,
      "relationship": 2
    }
  },
  {
```

```
"keys": [
  "source",
  "target",
  "relationship"
],
"length": 3,
"_fields": [
  {
    "identity": {
      "low": 29600,
      "high": 0
    },
    "labels": [
      "PharmacologicClass"
    ],
    "properties": {
      "license": "CC BY 4.0",
      "identifier": "N0000006277",
      "class_type": "Chemical/Ingredient",
      "name": "Vitamin D",
      "source": "FDA via DrugCentral",
      "url": "http://purl.bioontology.org/ontology/NDFRT/N0000006277"
    }
  },
  {
    "identity": {
      "low": 46428,
      "high": 0
    },
    "labels": [
      "Compound"
    ],
    "properties": {
      "license": "CC BY-NC 4.0",
      "identifier": "DB02300",
      "inchikey": "InChIKey=LWQQLNNNIPYSNX-UROSTWAQSA-N",
      "inchi": "InChI=1S/C27H40O3/c1-17(6-13-25(29)20-8-9-20)23-11-12-
      24-19(5-4-14-27(23,24)3)7-10-21-15-22(28)16-26(30)18(21)2/h6-7,
      10,13,17,20,22-26,28-30H,2,4-5,8-9,11-12,14-16H2,1,3H3/b13-6+,
      19-7+,21-10-/t17-,22-,23-,24+,25-,26+,27-/m1/s1",
      "name": "Calcipotriol",
      "source": "DrugBank",
      "url": "http://www.drugbank.ca/drugs/DB02300"
    }
  },
  {
    "identity": {
      "low": 1472934,
      "high": 0
    },
    "start": {
      "low": 29600,
      "high": 0
    },
    "end": {
      "low": 46428,
      "high": 0
    },
    "type": "INCLUDES_PCiC",
    "properties": {
      "license": "CC BY 4.0",
```

```
          "unbiased": false,
          "source": "DrugCentral"
        }
      }
    ],
    "_fieldLookup": {
      "source": 0,
      "target": 1,
      "relationship": 2
    }
  },
  {
    "keys": [
      "source",
      "target",
      "relationship"
    ],
    "length": 3,
    "_fields": [
      {
        "identity": {
          "low": 31332,
          "high": 0
        },
        "labels": [
          "Gene"
        ],
        "properties": {
          "license": "CC0 1.0",
          "identifier": {
            "low": 2104,
            "high": 0
          },
          "chromosome": "1",
          "name": "ESRRG",
          "description": "estrogen-related receptor gamma",
          "source": "Entrez Gene",
          "url": "http://identifiers.org/ncbigene/2104"
        }
      },
      {
        "identity": {
          "low": 18529,
          "high": 0
        },
        "labels": [
          "Gene"
        ],
        "properties": {
          "license": "CC0 1.0",
          "identifier": {
            "low": 23338,
            "high": 0
          },
          "chromosome": "5",
          "name": "JADE2",
          "description": "jade family PHD finger 2",
          "source": "Entrez Gene",
          "url": "http://identifiers.org/ncbigene/23338"
        }
      },
```

```
      {
        "identity": {
          "low": 231936,
          "high": 0
        },
        "start": {
          "low": 31332,
          "high": 0
        },
        "end": {
          "low": 18529,
          "high": 0
        },
        "type": "REGULATES_GrG",
        "properties": {
          "method": "measured",
          "unbiased": true,
          "source": "LINCS L1000",
          "subtypes": [
            "overexpression upregulates"
          ]
        }
      }
    ],
    "_fieldLookup": {
      "source": 0,
      "target": 1,
      "relationship": 2
    }
  },
  {
    "keys": [
      "source",
      "target",
      "relationship"
    ],
    "length": 3,
    "_fields": [
      {
        "identity": {
          "low": 46428,
          "high": 0
        },
        "labels": [
          "Compound"
        ],
        "properties": {
          "license": "CC BY-NC 4.0",
          "identifier": "DB02300",
          "inchikey": "InChIKey=LWQQLNNNIPYSNX-UROSTWAQSA-N",
          "inchi": "InChI=1S/C27H40O3/c1-17(6-13-25(29)20-8-9-20)23-11-12-
          24-19(5-4-14-27(23,24)3)7-10-21-15-22(28)16-26(30)18(21)2/h6-7,
          10,13,17,20,22-26,28-30H,2,4-5,8-9,11-12,14-16H2,1,3H3/b13-6+,
          19-7+,21-10-/t17-,22-,23-,24+,25-,26+,27-/m1/s1",
          "name": "Calcipotriol",
          "source": "DrugBank",
          "url": "http://www.drugbank.ca/drugs/DB02300"
        }
      },
      {
        "identity": {
```

```
          "low": 18529,
          "high": 0
        },
        "labels": [
          "Gene"
        ],
        "properties": {
          "license": "CC0 1.0",
          "identifier": {
            "low": 23338,
            "high": 0
          },
          "chromosome": "5",
          "name": "JADE2",
          "description": "jade family PHD finger 2",
          "source": "Entrez Gene",
          "url": "http://identifiers.org/ncbigene/23338"
        }
      },
      {
        "identity": {
          "low": 860335,
          "high": 0
        },
        "start": {
          "low": 46428,
          "high": 0
        },
        "end": {
          "low": 18529,
          "high": 0
        },
        "type": "DOWNREGULATES_CdG",
        "properties": {
          "method": "measured",
          "unbiased": true,
          "z_score": -4.533,
          "source": "LINCS L1000"
        }
      }
    ],
    "_fieldLookup": {
      "source": 0,
      "target": 1,
      "relationship": 2
    }
  },
  {
    "keys": [
      "source",
      "target",
      "relationship"
    ],
    "length": 3,
    "_fields": [
      {
        "identity": {
          "low": 31332,
          "high": 0
        },
        "labels": [
```

```
      "Gene"
    ],
    "properties": {
      "license": "CC0 1.0",
      "identifier": {
        "low": 2104,
        "high": 0
      },
      "chromosome": "1",
      "name": "ESRRG",
      "description": "estrogen-related receptor gamma",
      "source": "Entrez Gene",
      "url": "http://identifiers.org/ncbigene/2104"
    }
  },
  {
    "identity": {
      "low": 626,
      "high": 0
    },
    "labels": [
      "Gene"
    ],
    "properties": {
      "license": "CC0 1.0",
      "identifier": {
        "low": 51203,
        "high": 0
      },
      "chromosome": "15",
      "name": "NUSAP1",
      "description": "nucleolar and spindle associated protein 1",
      "source": "Entrez Gene",
      "url": "http://identifiers.org/ncbigene/51203"
    }
  },
  {
    "identity": {
      "low": 1248378,
      "high": 0
    },
    "start": {
      "low": 31332,
      "high": 0
    },
    "end": {
      "low": 626,
      "high": 0
    },
    "type": "REGULATES_GrG",
    "properties": {
      "method": "measured",
      "unbiased": true,
      "source": "LINCS L1000",
      "subtypes": [
        "overexpression downregulates"
      ]
    }
  }
],
"_fieldLookup": {
```

```
      "source": 0,
      "target": 1,
      "relationship": 2
    }
  },
  {
    "keys": [
      "source",
      "target",
      "relationship"
    ],
    "length": 3,
    "_fields": [
      {
        "identity": {
          "low": 46428,
          "high": 0
        },
        "labels": [
          "Compound"
        ],
        "properties": {
          "license": "CC BY-NC 4.0",
          "identifier": "DB02300",
          "inchikey": "InChIKey=LWQQLNNNIPYSNX-UROSTWAQSA-N",
          "inchi": "InChI=1S/C27H40O3/c1-17(6-13-25(29)20-8-9-20)23-11-12-
          24-19(5-4-14-27(23,24)3)7-10-21-15-22(28)16-26(30)18(21)2/h6-7,
          10,13,17,20,22-26,28-30H,2,4-5,8-9,11-12,14-16H2,1,3H3/b13-6+,
          19-7+,21-10-/t17-,22-,23-,24+,25-,26+,27-/m1/s1",
          "name": "Calcipotriol",
          "source": "DrugBank",
          "url": "http://www.drugbank.ca/drugs/DB02300"
        }
      },
      {
        "identity": {
          "low": 626,
          "high": 0
        },
        "labels": [
          "Gene"
        ],
        "properties": {
          "license": "CC0 1.0",
          "identifier": {
            "low": 51203,
            "high": 0
          },
          "chromosome": "15",
          "name": "NUSAP1",
          "description": "nucleolar and spindle associated protein 1",
          "source": "Entrez Gene",
          "url": "http://identifiers.org/ncbigene/51203"
        }
      },
      {
        "identity": {
          "low": 957014,
          "high": 0
        },
        "start": {
```

```
        "low": 46428,
        "high": 0
      },
      "end": {
        "low": 626,
        "high": 0
      },
      "type": "DOWNREGULATES_CdG",
      "properties": {
        "method": "measured",
        "unbiased": true,
        "z_score": -4.07,
        "source": "LINCS L1000"
      }
    }
  ],
  "_fieldLookup": {
    "source": 0,
    "target": 1,
    "relationship": 2
  }
},
{
  "keys": [
    "source",
    "target",
    "relationship"
  ],
  "length": 3,
  "_fields": [
    {
      "identity": {
        "low": 31332,
        "high": 0
      },
      "labels": [
        "Gene"
      ],
      "properties": {
        "license": "CC0 1.0",
        "identifier": {
          "low": 2104,
          "high": 0
        },
        "chromosome": "1",
        "name": "ESRRG",
        "description": "estrogen-related receptor gamma",
        "source": "Entrez Gene",
        "url": "http://identifiers.org/ncbigene/2104"
      }
    },
    {
      "identity": {
        "low": 36034,
        "high": 0
      },
      "labels": [
        "Gene"
      ],
      "properties": {
        "license": "CC0 1.0",
```

```
          "identifier": {
            "low": 3028,
            "high": 0
          },
          "chromosome": "X",
          "name": "HSD17B10",
          "description": "hydroxysteroid (17-beta) dehydrogenase 10",
          "source": "Entrez Gene",
          "url": "http://identifiers.org/ncbigene/3028"
        }
      },
      {
        "identity": {
          "low": 404166,
          "high": 0
        },
        "start": {
          "low": 31332,
          "high": 0
        },
        "end": {
          "low": 36034,
          "high": 0
        },
        "type": "REGULATES_GrG",
        "properties": {
          "method": "measured",
          "unbiased": true,
          "source": "LINCS L1000",
          "subtypes": [
            "overexpression upregulates"
          ]
        }
      }
    ],
    "_fieldLookup": {
      "source": 0,
      "target": 1,
      "relationship": 2
    }
  },
  {
    "keys": [
      "source",
      "target",
      "relationship"
    ],
    "length": 3,
    "_fields": [
      {
        "identity": {
          "low": 46428,
          "high": 0
        },
        "labels": [
          "Compound"
        ],
        "properties": {
          "license": "CC BY-NC 4.0",
          "identifier": "DB02300",
          "inchikey": "InChIKey=LWQQLNNNIPYSNX-UROSTWAQSA-N",
```

```
          "inchi": "InChI=1S/C27H40O3/c1-17(6-13-25(29)20-8-9-20)23-11-12-
          24-19(5-4-14-27(23,24)3)7-10-21-15-22(28)16-26(30)18(21)2/h6-7,
          10,13,17,20,22-26,28-30H,2,4-5,8-9,11-12,14-16H2,1,3H3/b13-6+,
          19-7+,21-10-/t17-,22-,23-,24+,25-,26+,27-/m1/s1",
          "name": "Calcipotriol",
          "source": "DrugBank",
          "url": "http://www.drugbank.ca/drugs/DB02300"
        }
      },
      {
        "identity": {
          "low": 36034,
          "high": 0
        },
        "labels": [
          "Gene"
        ],
        "properties": {
          "license": "CC0 1.0",
          "identifier": {
            "low": 3028,
            "high": 0
          },
          "chromosome": "X",
          "name": "HSD17B10",
          "description": "hydroxysteroid (17-beta) dehydrogenase 10",
          "source": "Entrez Gene",
          "url": "http://identifiers.org/ncbigene/3028"
        }
      },
      {
        "identity": {
          "low": 936945,
          "high": 0
        },
        "start": {
          "low": 46428,
          "high": 0
        },
        "end": {
          "low": 36034,
          "high": 0
        },
        "type": "DOWNREGULATES_CdG",
        "properties": {
          "method": "measured",
          "unbiased": true,
          "z_score": -4.664,
          "source": "LINCS L1000"
        }
      }
    ],
    "_fieldLookup": {
      "source": 0,
      "target": 1,
      "relationship": 2
    }
  },
  {
    "keys": [
      "source",
```

```
    "target",
    "relationship"
  ],
  "length": 3,
  "_fields": [
    {
      "identity": {
        "low": 31332,
        "high": 0
      },
      "labels": [
        "Gene"
      ],
      "properties": {
        "license": "CC0 1.0",
        "identifier": {
          "low": 2104,
          "high": 0
        },
        "chromosome": "1",
        "name": "ESRRG",
        "description": "estrogen-related receptor gamma",
        "source": "Entrez Gene",
        "url": "http://identifiers.org/ncbigene/2104"
      }
    },
    {
      "identity": {
        "low": 29588,
        "high": 0
      },
      "labels": [
        "Gene"
      ],
      "properties": {
        "license": "CC0 1.0",
        "identifier": {
          "low": 4864,
          "high": 0
        },
        "chromosome": "18",
        "name": "NPC1",
        "description": "Niemann-Pick disease, type C1",
        "source": "Entrez Gene",
        "url": "http://identifiers.org/ncbigene/4864"
      }
    },
    {
      "identity": {
        "low": 166428,
        "high": 0
      },
      "start": {
        "low": 31332,
        "high": 0
      },
      "end": {
        "low": 29588,
        "high": 0
      },
      "type": "REGULATES_GrG",
```

```
        "properties": {
          "method": "measured",
          "unbiased": true,
          "source": "LINCS L1000",
          "subtypes": [
            "overexpression upregulates"
          ]
        }
      }
    ],
    "_fieldLookup": {
      "source": 0,
      "target": 1,
      "relationship": 2
    }
  },
  {
    "keys": [
      "source",
      "target",
      "relationship"
    ],
    "length": 3,
    "_fields": [
      {
        "identity": {
          "low": 46428,
          "high": 0
        },
        "labels": [
          "Compound"
        ],
        "properties": {
          "license": "CC BY-NC 4.0",
          "identifier": "DB02300",
          "inchikey": "InChIKey=LWQQLNNNIPYSNX-UROSTWAQSA-N",
          "inchi": "InChI=1S/C27H40O3/c1-17(6-13-25(29)20-8-9-20)23-11-12-
          24-19(5-4-14-27(23,24)3)7-10-21-15-22(28)16-26(30)18(21)2/h6-7,
          10,13,17,20,22-26,28-30H,2,4-5,8-9,11-12,14-16H2,1,3H3/b13-6+,
          19-7+,21-10-/t17-,22-,23-,24+,25-,26+,27-/m1/s1",
          "name": "Calcipotriol",
          "source": "DrugBank",
          "url": "http://www.drugbank.ca/drugs/DB02300"
        }
      },
      {
        "identity": {
          "low": 29588,
          "high": 0
        },
        "labels": [
          "Gene"
        ],
        "properties": {
          "license": "CC0 1.0",
          "identifier": {
            "low": 4864,
            "high": 0
          },
          "chromosome": "18",
          "name": "NPC1",
```

```
          "description": "Niemann-Pick disease, type C1",
          "source": "Entrez Gene",
          "url": "http://identifiers.org/ncbigene/4864"
        }
      },
      {
        "identity": {
          "low": 460805,
          "high": 0
        },
        "start": {
          "low": 46428,
          "high": 0
        },
        "end": {
          "low": 29588,
          "high": 0
        },
        "type": "UPREGULATES_CuG",
        "properties": {
          "method": "measured",
          "unbiased": true,
          "z_score": 4.768,
          "source": "LINCS L1000"
        }
      }
    ],
    "_fieldLookup": {
      "source": 0,
      "target": 1,
      "relationship": 2
    }
  },
  {
    "keys": [
      "source",
      "target",
      "relationship"
    ],
    "length": 3,
    "_fields": [
      {
        "identity": {
          "low": 31332,
          "high": 0
        },
        "labels": [
          "Gene"
        ],
        "properties": {
          "license": "CC0 1.0",
          "identifier": {
            "low": 2104,
            "high": 0
          },
          "chromosome": "1",
          "name": "ESRRG",
          "description": "estrogen-related receptor gamma",
          "source": "Entrez Gene",
          "url": "http://identifiers.org/ncbigene/2104"
        }
```

```
      },
      {
        "identity": {
          "low": 887,
          "high": 0
        },
        "labels": [
          "Gene"
        ],
        "properties": {
          "license": "CC0 1.0",
          "identifier": {
            "low": 11098,
            "high": 0
          },
          "chromosome": "11",
          "name": "PRSS23",
          "description": "protease, serine, 23",
          "source": "Entrez Gene",
          "url": "http://identifiers.org/ncbigene/11098"
        }
      },
      {
        "identity": {
          "low": 1868836,
          "high": 0
        },
        "start": {
          "low": 31332,
          "high": 0
        },
        "end": {
          "low": 887,
          "high": 0
        },
        "type": "REGULATES_GrG",
        "properties": {
          "method": "measured",
          "unbiased": true,
          "source": "LINCS L1000",
          "subtypes": [
            "overexpression upregulates"
          ]
        }
      }
    ],
    "_fieldLookup": {
      "source": 0,
      "target": 1,
      "relationship": 2
    }
  },
  {
    "keys": [
      "source",
      "target",
      "relationship"
    ],
    "length": 3,
    "_fields": [
      {
```

```
  "identity": {
    "low": 46428,
    "high": 0
  },
  "labels": [
    "Compound"
  ],
  "properties": {
    "license": "CC BY-NC 4.0",
    "identifier": "DB02300",
    "inchikey": "InChIKey=LWQQLNNNIPYSNX-UROSTWAQSA-N",
    "inchi": "InChI=1S/C27H40O3/c1-17(6-13-25(29)20-8-9-20)23-11-12-
    24-19(5-4-14-27(23,24)3)7-10-21-15-22(28)16-26(30)18(21)2/h6-7,
    10,13,17,20,22-26,28-30H,2,4-5,8-9,11-12,14-16H2,1,3H3/b13-6+,
    19-7+,21-10-/t17-,22-,23-,24+,25-,26+,27-/m1/s1",
    "name": "Calcipotriol",
    "source": "DrugBank",
    "url": "http://www.drugbank.ca/drugs/DB02300"
  }
},
{
  "identity": {
    "low": 887,
    "high": 0
  },
  "labels": [
    "Gene"
  ],
  "properties": {
    "license": "CC0 1.0",
    "identifier": {
      "low": 11098,
      "high": 0
    },
    "chromosome": "11",
    "name": "PRSS23",
    "description": "protease, serine, 23",
    "source": "Entrez Gene",
    "url": "http://identifiers.org/ncbigene/11098"
  }
},
{
  "identity": {
    "low": 1842547,
    "high": 0
  },
  "start": {
    "low": 46428,
    "high": 0
  },
  "end": {
    "low": 887,
    "high": 0
  },
  "type": "DOWNREGULATES_CdG",
  "properties": {
    "method": "measured",
    "unbiased": true,
    "z_score": -5.937,
    "source": "LINCS L1000"
  }
```

```
      }
    ],
    "_fieldLookup": {
      "source": 0,
      "target": 1,
      "relationship": 2
    }
  },
  {
    "keys": [
      "source",
      "target",
      "relationship"
    ],
    "length": 3,
    "_fields": [
      {
        "identity": {
          "low": 31332,
          "high": 0
        },
        "labels": [
          "Gene"
        ],
        "properties": {
          "license": "CC0 1.0",
          "identifier": {
            "low": 2104,
            "high": 0
          },
          "chromosome": "1",
          "name": "ESRRG",
          "description": "estrogen-related receptor gamma",
          "source": "Entrez Gene",
          "url": "http://identifiers.org/ncbigene/2104"
        }
      },
      {
        "identity": {
          "low": 12074,
          "high": 0
        },
        "labels": [
          "Gene"
        ],
        "properties": {
          "license": "CC0 1.0",
          "identifier": {
            "low": 976,
            "high": 0
          },
          "chromosome": "19",
          "name": "ADGRE5",
          "description": "adhesion G protein-coupled receptor E5",
          "source": "Entrez Gene",
          "url": "http://identifiers.org/ncbigene/976"
        }
      },
      {
        "identity": {
          "low": 1857482,
```

```
            "high": 0
          },
          "start": {
            "low": 31332,
            "high": 0
          },
          "end": {
            "low": 12074,
            "high": 0
          },
          "type": "REGULATES_GrG",
          "properties": {
            "method": "measured",
            "unbiased": true,
            "source": "LINCS L1000",
            "subtypes": [
              "overexpression upregulates"
            ]
          }
        }
      ],
      "_fieldLookup": {
        "source": 0,
        "target": 1,
        "relationship": 2
      }
    },
    {
      "keys": [
        "source",
        "target",
        "relationship"
      ],
      "length": 3,
      "_fields": [
        {
          "identity": {
            "low": 46428,
            "high": 0
          },
          "labels": [
            "Compound"
          ],
          "properties": {
            "license": "CC BY-NC 4.0",
            "identifier": "DB02300",
            "inchikey": "InChIKey=LWQQLNNNIPYSNX-UROSTWAQSA-N",
            "inchi": "InChI=1S/C27H40O3/c1-17(6-13-25(29)20-8-9-20)23-11-12-
            24-19(5-4-14-27(23,24)3)7-10-21-15-22(28)16-26(30)18(21)2/h6-7,
            10,13,17,20,22-26,28-30H,2,4-5,8-9,11-12,14-16H2,1,3H3/b13-6+,
            19-7+,21-10-/t17-,22-,23-,24+,25-,26+,27-/m1/s1",
            "name": "Calcipotriol",
            "source": "DrugBank",
            "url": "http://www.drugbank.ca/drugs/DB02300"
          }
        },
        {
          "identity": {
            "low": 12074,
            "high": 0
          },
```

```
        "labels": [
          "Gene"
        ],
        "properties": {
          "license": "CC0 1.0",
          "identifier": {
            "low": 976,
            "high": 0
          },
          "chromosome": "19",
          "name": "ADGRE5",
          "description": "adhesion G protein-coupled receptor E5",
          "source": "Entrez Gene",
          "url": "http://identifiers.org/ncbigene/976"
        }
      },
      {
        "identity": {
          "low": 1854319,
          "high": 0
        },
        "start": {
          "low": 46428,
          "high": 0
        },
        "end": {
          "low": 12074,
          "high": 0
        },
        "type": "UPREGULATES_CuG",
        "properties": {
          "method": "measured",
          "unbiased": true,
          "z_score": 6.13,
          "source": "LINCS L1000"
        }
      }
    ],
    "_fieldLookup": {
      "source": 0,
      "target": 1,
      "relationship": 2
    }
  },
  {
    "keys": [
      "source",
      "target",
      "relationship"
    ],
    "length": 3,
    "_fields": [
      {
        "identity": {
          "low": 31332,
          "high": 0
        },
        "labels": [
          "Gene"
        ],
        "properties": {
```

```
      "license": "CC0 1.0",
      "identifier": {
        "low": 2104,
        "high": 0
      },
      "chromosome": "1",
      "name": "ESRRG",
      "description": "estrogen-related receptor gamma",
      "source": "Entrez Gene",
      "url": "http://identifiers.org/ncbigene/2104"
    }
  },
  {
    "identity": {
      "low": 33164,
      "high": 0
    },
    "labels": [
      "Gene"
    ],
    "properties": {
      "license": "CC0 1.0",
      "identifier": {
        "low": 5154,
        "high": 0
      },
      "chromosome": "7",
      "name": "PDGFA",
      "description": "platelet-derived growth factor alpha polypeptide",
      "source": "Entrez Gene",
      "url": "http://identifiers.org/ncbigene/5154"
    }
  },
  {
    "identity": {
      "low": 346975,
      "high": 0
    },
    "start": {
      "low": 31332,
      "high": 0
    },
    "end": {
      "low": 33164,
      "high": 0
    },
    "type": "REGULATES_GrG",
    "properties": {
      "method": "measured",
      "unbiased": true,
      "source": "LINCS L1000",
      "subtypes": [
        "overexpression upregulates"
      ]
    }
  }
],
"_fieldLookup": {
  "source": 0,
  "target": 1,
  "relationship": 2
```

```
    }
  },
  {
    "keys": [
      "source",
      "target",
      "relationship"
    ],
    "length": 3,
    "_fields": [
      {
        "identity": {
          "low": 46428,
          "high": 0
        },
        "labels": [
          "Compound"
        ],
        "properties": {
          "license": "CC BY-NC 4.0",
          "identifier": "DB02300",
          "inchikey": "InChIKey=LWQQLNNNIPYSNX-UROSTWAQSA-N",
          "inchi": "InChI=1S/C27H40O3/c1-17(6-13-25(29)20-8-9-20)23-11-12-
          24-19(5-4-14-27(23,24)3)7-10-21-15-22(28)16-26(30)18(21)2/h6-7,
          10,13,17,20,22-26,28-30H,2,4-5,8-9,11-12,14-16H2,1,3H3/b13-6+,
          19-7+,21-10-/t17-,22-,23-,24+,25-,26+,27-/m1/s1",
          "name": "Calcipotriol",
          "source": "DrugBank",
          "url": "http://www.drugbank.ca/drugs/DB02300"
        }
      },
      {
        "identity": {
          "low": 33164,
          "high": 0
        },
        "labels": [
          "Gene"
        ],
        "properties": {
          "license": "CC0 1.0",
          "identifier": {
            "low": 5154,
            "high": 0
          },
          "chromosome": "7",
          "name": "PDGFA",
          "description": "platelet-derived growth factor alpha polypeptide",
          "source": "Entrez Gene",
          "url": "http://identifiers.org/ncbigene/5154"
        }
      },
      {
        "identity": {
          "low": 1600138,
          "high": 0
        },
        "start": {
          "low": 46428,
          "high": 0
        },
```

```
        "end": {
          "low": 33164,
          "high": 0
        },
        "type": "UPREGULATES_CuG",
        "properties": {
          "method": "measured",
          "unbiased": true,
          "z_score": 4.66,
          "source": "LINCS L1000"
        }
      }
    ],
    "_fieldLookup": {
      "source": 0,
      "target": 1,
      "relationship": 2
    }
  },
  {
    "keys": [
      "source",
      "target",
      "relationship"
    ],
    "length": 3,
    "_fields": [
      {
        "identity": {
          "low": 31332,
          "high": 0
        },
        "labels": [
          "Gene"
        ],
        "properties": {
          "license": "CC0 1.0",
          "identifier": {
            "low": 2104,
            "high": 0
          },
          "chromosome": "1",
          "name": "ESRRG",
          "description": "estrogen-related receptor gamma",
          "source": "Entrez Gene",
          "url": "http://identifiers.org/ncbigene/2104"
        }
      },
      {
        "identity": {
          "low": 24126,
          "high": 0
        },
        "labels": [
          "Gene"
        ],
        "properties": {
          "license": "CC0 1.0",
          "identifier": {
            "low": 3486,
            "high": 0
```

```
          },
          "chromosome": "7",
          "name": "IGFBP3",
          "description": "insulin-like growth factor binding protein 3",
          "source": "Entrez Gene",
          "url": "http://identifiers.org/ncbigene/3486"
        }
      },
      {
        "identity": {
          "low": 253555,
          "high": 0
        },
        "start": {
          "low": 31332,
          "high": 0
        },
        "end": {
          "low": 24126,
          "high": 0
        },
        "type": "REGULATES_GrG",
        "properties": {
          "method": "measured",
          "unbiased": true,
          "source": "LINCS L1000",
          "subtypes": [
            "overexpression downregulates"
          ]
        }
      }
    ],
    "_fieldLookup": {
      "source": 0,
      "target": 1,
      "relationship": 2
    }
  },
  {
    "keys": [
      "source",
      "target",
      "relationship"
    ],
    "length": 3,
    "_fields": [
      {
        "identity": {
          "low": 46428,
          "high": 0
        },
        "labels": [
          "Compound"
        ],
        "properties": {
          "license": "CC BY-NC 4.0",
          "identifier": "DB02300",
          "inchikey": "InChIKey=LWQQLNNNIPYSNX-UROSTWAQSA-N",
          "inchi": "InChI=1S/C27H40O3/c1-17(6-13-25(29)20-8-9-20)23-11-12-
          24-19(5-4-14-27(23,24)3)7-10-21-15-22(28)16-26(30)18(21)2/h6-7,
          10,13,17,20,22-26,28-30H,2,4-5,8-9,11-12,14-16H2,1,3H3/b13-6+,
```

```
        19-7+,21-10-/t17-,22-,23-,24+,25-,26+,27-/m1/s1",
        "name": "Calcipotriol",
        "source": "DrugBank",
        "url": "http://www.drugbank.ca/drugs/DB02300"
      }
    },
    {
      "identity": {
        "low": 24126,
        "high": 0
      },
      "labels": [
        "Gene"
      ],
      "properties": {
        "license": "CC0 1.0",
        "identifier": {
          "low": 3486,
          "high": 0
        },
        "chromosome": "7",
        "name": "IGFBP3",
        "description": "insulin-like growth factor binding protein 3",
        "source": "Entrez Gene",
        "url": "http://identifiers.org/ncbigene/3486"
      }
    },
    {
      "identity": {
        "low": 1433722,
        "high": 0
      },
      "start": {
        "low": 46428,
        "high": 0
      },
      "end": {
        "low": 24126,
        "high": 0
      },
      "type": "UPREGULATES_CuG",
      "properties": {
        "method": "measured",
        "unbiased": true,
        "z_score": 7.768,
        "source": "LINCS L1000"
      }
    }
  ],
  "_fieldLookup": {
    "source": 0,
    "target": 1,
    "relationship": 2
  }
},
{
  "keys": [
    "source",
    "target",
    "relationship"
  ],
```

```
      "length": 3,
      "_fields": [
        {
          "identity": {
            "low": 31332,
            "high": 0
          },
          "labels": [
            "Gene"
          ],
          "properties": {
            "license": "CC0 1.0",
            "identifier": {
              "low": 2104,
              "high": 0
            },
            "chromosome": "1",
            "name": "ESRRG",
            "description": "estrogen-related receptor gamma",
            "source": "Entrez Gene",
            "url": "http://identifiers.org/ncbigene/2104"
          }
        },
        {
          "identity": {
            "low": 43663,
            "high": 0
          },
          "labels": [
            "Gene"
          ],
          "properties": {
            "license": "CC0 1.0",
            "identifier": {
              "low": 230,
              "high": 0
            },
            "chromosome": "17",
            "name": "ALDOC",
            "description": "aldolase C, fructose-bisphosphate",
            "source": "Entrez Gene",
            "url": "http://identifiers.org/ncbigene/230"
          }
        },
        {
          "identity": {
            "low": 2222777,
            "high": 0
          },
          "start": {
            "low": 31332,
            "high": 0
          },
          "end": {
            "low": 43663,
            "high": 0
          },
          "type": "REGULATES_GrG",
          "properties": {
            "method": "measured",
            "unbiased": true,
```

```
          "source": "LINCS L1000",
          "subtypes": [
            "overexpression upregulates"
          ]
        }
      }
    ],
    "_fieldLookup": {
      "source": 0,
      "target": 1,
      "relationship": 2
    }
  },
  {
    "keys": [
      "source",
      "target",
      "relationship"
    ],
    "length": 3,
    "_fields": [
      {
        "identity": {
          "low": 46428,
          "high": 0
        },
        "labels": [
          "Compound"
        ],
        "properties": {
          "license": "CC BY-NC 4.0",
          "identifier": "DB02300",
          "inchikey": "InChIKey=LWQQLNNNIPYSNX-UROSTWAQSA-N",
          "inchi": "InChI=1S/C27H40O3/c1-17(6-13-25(29)20-8-9-20)23-11-12-
          24-19(5-4-14-27(23,24)3)7-10-21-15-22(28)16-26(30)18(21)2/h6-7,
          10,13,17,20,22-26,28-30H,2,4-5,8-9,11-12,14-16H2,1,3H3/b13-6+,
          19-7+,21-10-/t17-,22-,23-,24+,25-,26+,27-/m1/s1",
          "name": "Calcipotriol",
          "source": "DrugBank",
          "url": "http://www.drugbank.ca/drugs/DB02300"
        }
      },
      {
        "identity": {
          "low": 43663,
          "high": 0
        },
        "labels": [
          "Gene"
        ],
        "properties": {
          "license": "CC0 1.0",
          "identifier": {
            "low": 230,
            "high": 0
          },
          "chromosome": "17",
          "name": "ALDOC",
          "description": "aldolase C, fructose-bisphosphate",
          "source": "Entrez Gene",
          "url": "http://identifiers.org/ncbigene/230"
```

```
        }
      },
      {
        "identity": {
          "low": 360626,
          "high": 0
        },
        "start": {
          "low": 46428,
          "high": 0
        },
        "end": {
          "low": 43663,
          "high": 0
        },
        "type": "UPREGULATES_CuG",
        "properties": {
          "method": "measured",
          "unbiased": true,
          "z_score": 4.822,
          "source": "LINCS L1000"
        }
      }
    ],
    "_fieldLookup": {
      "source": 0,
      "target": 1,
      "relationship": 2
    }
  },
  {
    "keys": [
      "source",
      "target",
      "relationship"
    ],
    "length": 3,
    "_fields": [
      {
        "identity": {
          "low": 31332,
          "high": 0
        },
        "labels": [
          "Gene"
        ],
        "properties": {
          "license": "CC0 1.0",
          "identifier": {
            "low": 2104,
            "high": 0
          },
          "chromosome": "1",
          "name": "ESRRG",
          "description": "estrogen-related receptor gamma",
          "source": "Entrez Gene",
          "url": "http://identifiers.org/ncbigene/2104"
        }
      },
      {
        "identity": {
```

```
          "low": 43712,
          "high": 0
        },
        "labels": [
          "Gene"
        ],
        "properties": {
          "license": "CC0 1.0",
          "identifier": {
            "low": 64943,
            "high": 0
          },
          "chromosome": "3",
          "name": "NT5DC2",
          "description": "5’-nucleotidase domain containing 2",
          "source": "Entrez Gene",
          "url": "http://identifiers.org/ncbigene/64943"
        }
      },
      {
        "identity": {
          "low": 1066423,
          "high": 0
        },
        "start": {
          "low": 31332,
          "high": 0
        },
        "end": {
          "low": 43712,
          "high": 0
        },
        "type": "REGULATES_GrG",
        "properties": {
          "method": "measured",
          "unbiased": true,
          "source": "LINCS L1000",
          "subtypes": [
            "overexpression downregulates"
          ]
        }
      }
    ],
    "_fieldLookup": {
      "source": 0,
      "target": 1,
      "relationship": 2
    }
  },
  {
    "keys": [
      "source",
      "target",
      "relationship"
    ],
    "length": 3,
    "_fields": [
      {
        "identity": {
          "low": 26360,
          "high": 0
```

```
      },
      "labels": [
        "Compound"
      ],
      "properties": {
        "license": "CC BY-NC 4.0",
        "identifier": "DB00162",
        "inchikey": "InChIKey=FPIPGXGPPPQFEQ-OVSJKPMPSA-N",
        "inchi": "InChI=1S/C20H30O/c1-16(8-6-9-17(2)13-15-21)11-12-19-18(3)
        10-7-14-20(19,4)5/h6,8-9,11-13,21H,7,10,14-15H2,1-5H3/b9-6+,12-11+,
        16-8+,17-13+",
        "name": "Vitamin A",
        "source": "DrugBank",
        "url": "http://www.drugbank.ca/drugs/DB00162"
      }
    },
    {
      "identity": {
        "low": 43712,
        "high": 0
      },
      "labels": [
        "Gene"
      ],
      "properties": {
        "license": "CC0 1.0",
        "identifier": {
          "low": 64943,
          "high": 0
        },
        "chromosome": "3",
        "name": "NT5DC2",
        "description": "5'-nucleotidase domain containing 2",
        "source": "Entrez Gene",
        "url": "http://identifiers.org/ncbigene/64943"
      }
    },
    {
      "identity": {
        "low": 71087,
        "high": 0
      },
      "start": {
        "low": 26360,
        "high": 0
      },
      "end": {
        "low": 43712,
        "high": 0
      },
      "type": "DOWNREGULATES_CdG",
      "properties": {
        "method": "measured",
        "unbiased": true,
        "z_score": -5.164,
        "source": "LINCS L1000"
      }
    }
  ],
  "_fieldLookup": {
    "source": 0,
```

```
      "target": 1,
      "relationship": 2
    }
  },
  {
    "keys": [
      "source",
      "target",
      "relationship"
    ],
    "length": 3,
    "_fields": [
      {
        "identity": {
          "low": 37072,
          "high": 0
        },
        "labels": [
          "PharmacologicClass"
        ],
        "properties": {
          "license": "CC BY 4.0",
          "identifier": "N0000006269",
          "class_type": "Chemical/Ingredient",
          "name": "Vitamin A",
          "source": "FDA via DrugCentral",
          "url": "http://purl.bioontology.org/ontology/NDFRT/N0000006269"
        }
      },
      {
        "identity": {
          "low": 26360,
          "high": 0
        },
        "labels": [
          "Compound"
        ],
        "properties": {
          "license": "CC BY-NC 4.0",
          "identifier": "DB00162",
          "inchikey": "InChIKey=FPIPGXGPPPQFEQ-OVSJKPMPSA-N",
          "inchi": "InChI=1S/C20H30O/c1-16(8-6-9-17(2)13-15-21)11-12-19-18(3)
          10-7-14-20(19,4)5/h6,8-9,11-13,21H,7,10,14-15H2,1-5H3/b9-6+,12-11+,
          16-8+,17-13+",
          "name": "Vitamin A",
          "source": "DrugBank",
          "url": "http://www.drugbank.ca/drugs/DB00162"
        }
      },
      {
        "identity": {
          "low": 206064,
          "high": 0
        },
        "start": {
          "low": 37072,
          "high": 0
        },
        "end": {
          "low": 26360,
          "high": 0
```

```
        },
        "type": "INCLUDES_PCiC",
        "properties": {
          "license": "CC BY 4.0",
          "unbiased": false,
          "source": "DrugCentral"
        }
      }
    ],
    "_fieldLookup": {
      "source": 0,
      "target": 1,
      "relationship": 2
    }
  },
  {
    "keys": [
      "source",
      "target",
      "relationship"
    ],
    "length": 3,
    "_fields": [
      {
        "identity": {
          "low": 26360,
          "high": 0
        },
        "labels": [
          "Compound"
        ],
        "properties": {
          "license": "CC BY-NC 4.0",
          "identifier": "DB00162",
          "inchikey": "InChIKey=FPIPGXGPPPQFEQ-OVSJKPMPSA-N",
          "inchi": "InChI=1S/C20H30O/c1-16(8-6-9-17(2)13-15-21)11-12-19-18(3)
          10-7-14-20(19,4)5/h6,8-9,11-13,21H,7,10,14-15H2,1-5H3/b9-6+,12-11+,
          16-8+,17-13+",
          "name": "Vitamin A",
          "source": "DrugBank",
          "url": "http://www.drugbank.ca/drugs/DB00162"
        }
      },
      {
        "identity": {
          "low": 24096,
          "high": 0
        },
        "labels": [
          "Gene"
        ],
        "properties": {
          "license": "CC0 1.0",
          "identifier": {
            "low": 3162,
            "high": 0
          },
          "chromosome": "22",
          "name": "HMOX1",
          "description": "heme oxygenase 1",
          "source": "Entrez Gene",
```

```
          "url": "http://identifiers.org/ncbigene/3162"
        }
      },
      {
        "identity": {
          "low": 325801,
          "high": 0
        },
        "start": {
          "low": 26360,
          "high": 0
        },
        "end": {
          "low": 24096,
          "high": 0
        },
        "type": "UPREGULATES_CuG",
        "properties": {
          "method": "measured",
          "unbiased": true,
          "z_score": 21.882,
          "source": "LINCS L1000"
        }
      }
    ],
    "_fieldLookup": {
      "source": 0,
      "target": 1,
      "relationship": 2
    }
  },
  {
    "keys": [
      "source",
      "target",
      "relationship"
    ],
    "length": 3,
    "_fields": [
      {
        "identity": {
          "low": 31332,
          "high": 0
        },
        "labels": [
          "Gene"
        ],
        "properties": {
          "license": "CC0 1.0",
          "identifier": {
            "low": 2104,
            "high": 0
          },
          "chromosome": "1",
          "name": "ESRRG",
          "description": "estrogen-related receptor gamma",
          "source": "Entrez Gene",
          "url": "http://identifiers.org/ncbigene/2104"
        }
      },
      {
```

```
        "identity": {
          "low": 8145,
          "high": 0
        },
        "labels": [
          "Gene"
        ],
        "properties": {
          "license": "CC0 1.0",
          "identifier": {
            "low": 4925,
            "high": 0
          },
          "chromosome": "11",
          "name": "NUCB2",
          "description": "nucleobindin 2",
          "source": "Entrez Gene",
          "url": "http://identifiers.org/ncbigene/4925"
        }
      },
      {
        "identity": {
          "low": 1209297,
          "high": 0
        },
        "start": {
          "low": 31332,
          "high": 0
        },
        "end": {
          "low": 8145,
          "high": 0
        },
        "type": "REGULATES_GrG",
        "properties": {
          "method": "measured",
          "unbiased": true,
          "source": "LINCS L1000",
          "subtypes": [
            "overexpression downregulates"
          ]
        }
      }
    ],
    "_fieldLookup": {
      "source": 0,
      "target": 1,
      "relationship": 2
    }
  },
  {
    "keys": [
      "source",
      "target",
      "relationship"
    ],
    "length": 3,
    "_fields": [
      {
        "identity": {
          "low": 26360,
```

```
        "high": 0
      },
      "labels": [
        "Compound"
      ],
      "properties": {
        "license": "CC BY-NC 4.0",
        "identifier": "DB00162",
        "inchikey": "InChIKey=FPIPGXGPPPQFEQ-OVSJKPMPSA-N",
        "inchi": "InChI=1S/C20H30O/c1-16(8-6-9-17(2)13-15-21)11-12-19-18(3)
        10-7-14-20(19,4)5/h6,8-9,11-13,21H,7,10,14-15H2,1-5H3/b9-6+,12-11+,
        16-8+,17-13+",
        "name": "Vitamin A",
        "source": "DrugBank",
        "url": "http://www.drugbank.ca/drugs/DB00162"
      }
    },
    {
      "identity": {
        "low": 8145,
        "high": 0
      },
      "labels": [
        "Gene"
      ],
      "properties": {
        "license": "CC0 1.0",
        "identifier": {
          "low": 4925,
          "high": 0
        },
        "chromosome": "11",
        "name": "NUCB2",
        "description": "nucleobindin 2",
        "source": "Entrez Gene",
        "url": "http://identifiers.org/ncbigene/4925"
      }
    },
    {
      "identity": {
        "low": 1060542,
        "high": 0
      },
      "start": {
        "low": 26360,
        "high": 0
      },
      "end": {
        "low": 8145,
        "high": 0
      },
      "type": "DOWNREGULATES_CdG",
      "properties": {
        "method": "measured",
        "unbiased": true,
        "z_score": -5.461,
        "source": "LINCS L1000"
      }
    }
  ],
  "_fieldLookup": {
```

```
        "source": 0,
        "target": 1,
        "relationship": 2
      }
    },
    {
      "keys": [
        "source",
        "target",
        "relationship"
      ],
      "length": 3,
      "_fields": [
        {
          "identity": {
            "low": 31332,
            "high": 0
          },
          "labels": [
            "Gene"
          ],
          "properties": {
            "license": "CC0 1.0",
            "identifier": {
              "low": 2104,
              "high": 0
            },
            "chromosome": "1",
            "name": "ESRRG",
            "description": "estrogen-related receptor gamma",
            "source": "Entrez Gene",
            "url": "http://identifiers.org/ncbigene/2104"
          }
        },
        {
          "identity": {
            "low": 40289,
            "high": 0
          },
          "labels": [
            "Gene"
          ],
          "properties": {
            "license": "CC0 1.0",
            "identifier": {
              "low": 6714,
              "high": 0
            },
            "chromosome": "20",
            "name": "SRC",
            "description": "SRC proto-oncogene, non-receptor tyrosine kinase",
            "source": "Entrez Gene",
            "url": "http://identifiers.org/ncbigene/6714"
          }
        },
        {
          "identity": {
            "low": 730995,
            "high": 0
          },
          "start": {
```

```
          "low": 31332,
          "high": 0
        },
        "end": {
          "low": 40289,
          "high": 0
        },
        "type": "INTERACTS_GiG",
        "properties": {
          "sources": [
            "hetio-dag"
          ],
          "unbiased": false
        }
      }
    ],
    "_fieldLookup": {
      "source": 0,
      "target": 1,
      "relationship": 2
    }
  },
  {
    "keys": [
      "source",
      "target",
      "relationship"
    ],
    "length": 3,
    "_fields": [
      {
        "identity": {
          "low": 26360,
          "high": 0
        },
        "labels": [
          "Compound"
        ],
        "properties": {
          "license": "CC BY-NC 4.0",
          "identifier": "DB00162",
          "inchikey": "InChIKey=FPIPGXGPPPQFEQ-OVSJKPMPSA-N",
          "inchi": "InChI=1S/C20H30O/c1-16(8-6-9-17(2)13-15-21)11-12-19-18(3)
          10-7-14-20(19,4)5/h6,8-9,11-13,21H,7,10,14-15H2,1-5H3/b9-6+,12-11+,
          16-8+,17-13+",
          "name": "Vitamin A",
          "source": "DrugBank",
          "url": "http://www.drugbank.ca/drugs/DB00162"
        }
      },
      {
        "identity": {
          "low": 40289,
          "high": 0
        },
        "labels": [
          "Gene"
        ],
        "properties": {
          "license": "CC0 1.0",
          "identifier": {
```

```
          "low": 6714,
          "high": 0
        },
        "chromosome": "20",
        "name": "SRC",
        "description": "SRC proto-oncogene, non-receptor tyrosine kinase",
        "source": "Entrez Gene",
        "url": "http://identifiers.org/ncbigene/6714"
      }
    },
    {
      "identity": {
        "low": 577290,
        "high": 0
      },
      "start": {
        "low": 26360,
        "high": 0
      },
      "end": {
        "low": 40289,
        "high": 0
      },
      "type": "UPREGULATES_CuG",
      "properties": {
        "method": "measured",
        "unbiased": true,
        "z_score": 5.408,
        "source": "LINCS L1000"
      }
    }
  ],
  "_fieldLookup": {
    "source": 0,
    "target": 1,
    "relationship": 2
  }
},
{
  "keys": [
    "source",
    "target",
    "relationship"
  ],
  "length": 3,
  "_fields": [
    {
      "identity": {
        "low": 31332,
        "high": 0
      },
      "labels": [
        "Gene"
      ],
      "properties": {
        "license": "CC0 1.0",
        "identifier": {
          "low": 2104,
          "high": 0
        },
        "chromosome": "1",
```

```
        "name": "ESRRG",
        "description": "estrogen-related receptor gamma",
        "source": "Entrez Gene",
        "url": "http://identifiers.org/ncbigene/2104"
      }
    },
    {
      "identity": {
        "low": 45699,
        "high": 0
      },
      "labels": [
        "Gene"
      ],
      "properties": {
        "license": "CC0 1.0",
        "identifier": {
          "low": 9903,
          "high": 0
        },
        "chromosome": "1",
        "name": "KLHL21",
        "description": "kelch-like family member 21",
        "source": "Entrez Gene",
        "url": "http://identifiers.org/ncbigene/9903"
      }
    },
    {
      "identity": {
        "low": 1640663,
        "high": 0
      },
      "start": {
        "low": 31332,
        "high": 0
      },
      "end": {
        "low": 45699,
        "high": 0
      },
      "type": "REGULATES_GrG",
      "properties": {
        "method": "measured",
        "unbiased": true,
        "source": "LINCS L1000",
        "subtypes": [
          "overexpression upregulates"
        ]
      }
    }
  ],
  "_fieldLookup": {
    "source": 0,
    "target": 1,
    "relationship": 2
  }
},
{
  "keys": [
    "source",
    "target",
```

```
  "relationship"
],
"length": 3,
"_fields": [
  {
    "identity": {
      "low": 26360,
      "high": 0
    },
    "labels": [
      "Compound"
    ],
    "properties": {
      "license": "CC BY-NC 4.0",
      "identifier": "DB00162",
      "inchikey": "InChIKey=FPIPGXGPPPQFEQ-OVSJKPMPSA-N",
      "inchi": "InChI=1S/C20H30O/c1-16(8-6-9-17(2)13-15-21)11-12-19-18(3)
      10-7-14-20(19,4)5/h6,8-9,11-13,21H,7,10,14-15H2,1-5H3/b9-6+,12-11+,
      16-8+,17-13+",
      "name": "Vitamin A",
      "source": "DrugBank",
      "url": "http://www.drugbank.ca/drugs/DB00162"
    }
  },
  {
    "identity": {
      "low": 45699,
      "high": 0
    },
    "labels": [
      "Gene"
    ],
    "properties": {
      "license": "CC0 1.0",
      "identifier": {
        "low": 9903,
        "high": 0
      },
      "chromosome": "1",
      "name": "KLHL21",
      "description": "kelch-like family member 21",
      "source": "Entrez Gene",
      "url": "http://identifiers.org/ncbigene/9903"
    }
  },
  {
    "identity": {
      "low": 1718765,
      "high": 0
    },
    "start": {
      "low": 26360,
      "high": 0
    },
    "end": {
      "low": 45699,
      "high": 0
    },
    "type": "UPREGULATES_CuG",
    "properties": {
      "method": "measured",
```

```
          "unbiased": true,
          "z_score": 6.547,
          "source": "LINCS L1000"
        }
      }
    ],
    "_fieldLookup": {
      "source": 0,
      "target": 1,
      "relationship": 2
    }
  },
  {
    "keys": [
      "source",
      "target",
      "relationship"
    ],
    "length": 3,
    "_fields": [
      {
        "identity": {
          "low": 31332,
          "high": 0
        },
        "labels": [
          "Gene"
        ],
        "properties": {
          "license": "CC0 1.0",
          "identifier": {
            "low": 2104,
            "high": 0
          },
          "chromosome": "1",
          "name": "ESRRG",
          "description": "estrogen-related receptor gamma",
          "source": "Entrez Gene",
          "url": "http://identifiers.org/ncbigene/2104"
        }
      },
      {
        "identity": {
          "low": 21860,
          "high": 0
        },
        "labels": [
          "Gene"
        ],
        "properties": {
          "license": "CC0 1.0",
          "identifier": {
            "low": 8204,
            "high": 0
          },
          "chromosome": "21",
          "name": "NRIP1",
          "description": "nuclear receptor interacting protein 1",
          "source": "Entrez Gene",
          "url": "http://identifiers.org/ncbigene/8204"
        }
```

```
      },
      {
        "identity": {
          "low": 1867272,
          "high": 0
        },
        "start": {
          "low": 31332,
          "high": 0
        },
        "end": {
          "low": 21860,
          "high": 0
        },
        "type": "INTERACTS_GiG",
        "properties": {
          "sources": [
            "Lit-BM-13",
            "hetio-dag"
          ],
          "unbiased": false
        }
      }
    ],
    "_fieldLookup": {
      "source": 0,
      "target": 1,
      "relationship": 2
    }
  },
  {
    "keys": [
      "source",
      "target",
      "relationship"
    ],
    "length": 3,
    "_fields": [
      {
        "identity": {
          "low": 26360,
          "high": 0
        },
        "labels": [
          "Compound"
        ],
        "properties": {
          "license": "CC BY-NC 4.0",
          "identifier": "DB00162",
          "inchikey": "InChIKey=FPIPGXGPPPQFEQ-OVSJKPMPSA-N",
          "inchi": "InChI=1S/C20H30O/c1-16(8-6-9-17(2)13-15-21)11-12-19-18(3)
          10-7-14-20(19,4)5/h6,8-9,11-13,21H,7,10,14-15H2,1-5H3/b9-6+,12-11+,
          16-8+,17-13+",
          "name": "Vitamin A",
          "source": "DrugBank",
          "url": "http://www.drugbank.ca/drugs/DB00162"
        }
      },
      {
        "identity": {
          "low": 21860,
```

```
          "high": 0
        },
        "labels": [
          "Gene"
        ],
        "properties": {
          "license": "CC0 1.0",
          "identifier": {
            "low": 8204,
            "high": 0
          },
          "chromosome": "21",
          "name": "NRIP1",
          "description": "nuclear receptor interacting protein 1",
          "source": "Entrez Gene",
          "url": "http://identifiers.org/ncbigene/8204"
        }
      },
      {
        "identity": {
          "low": 269722,
          "high": 0
        },
        "start": {
          "low": 26360,
          "high": 0
        },
        "end": {
          "low": 21860,
          "high": 0
        },
        "type": "UPREGULATES_CuG",
        "properties": {
          "method": "measured",
          "unbiased": true,
          "z_score": 4.738,
          "source": "LINCS L1000"
        }
      }
    ],
    "_fieldLookup": {
      "source": 0,
      "target": 1,
      "relationship": 2
    }
  },
  {
    "keys": [
      "source",
      "target",
      "relationship"
    ],
    "length": 3,
    "_fields": [
      {
        "identity": {
          "low": 1204,
          "high": 0
        },
        "labels": [
          "Gene"
```

```
    ],
    "properties": {
      "license": "CC0 1.0",
      "identifier": {
        "low": 1616,
        "high": 0
      },
      "chromosome": "6",
      "name": "DAXX",
      "description": "death-domain associated protein",
      "source": "Entrez Gene",
      "url": "http://identifiers.org/ncbigene/1616"
    }
  },
  {
    "identity": {
      "low": 31332,
      "high": 0
    },
    "labels": [
      "Gene"
    ],
    "properties": {
      "license": "CC0 1.0",
      "identifier": {
        "low": 2104,
        "high": 0
      },
      "chromosome": "1",
      "name": "ESRRG",
      "description": "estrogen-related receptor gamma",
      "source": "Entrez Gene",
      "url": "http://identifiers.org/ncbigene/2104"
    }
  },
  {
    "identity": {
      "low": 1736126,
      "high": 0
    },
    "start": {
      "low": 1204,
      "high": 0
    },
    "end": {
      "low": 31332,
      "high": 0
    },
    "type": "INTERACTS_GiG",
    "properties": {
      "sources": [
        "hetio-dag"
      ],
      "unbiased": false
    }
  }
],
"_fieldLookup": {
  "source": 0,
  "target": 1,
  "relationship": 2
```

```
    }
  },
  {
    "keys": [
      "source",
      "target",
      "relationship"
    ],
    "length": 3,
    "_fields": [
      {
        "identity": {
          "low": 26360,
          "high": 0
        },
        "labels": [
          "Compound"
        ],
        "properties": {
          "license": "CC BY-NC 4.0",
          "identifier": "DB00162",
          "inchikey": "InChIKey=FPIPGXGPPPQFEQ-OVSJKPMPSA-N",
          "inchi": "InChI=1S/C20H30O/c1-16(8-6-9-17(2)13-15-21)11-12-19-18(3)
          10-7-14-20(19,4)5/h6,8-9,11-13,21H,7,10,14-15H2,1-5H3/b9-6+,12-11+,
          16-8+,17-13+",
          "name": "Vitamin A",
          "source": "DrugBank",
          "url": "http://www.drugbank.ca/drugs/DB00162"
        }
      },
      {
        "identity": {
          "low": 1204,
          "high": 0
        },
        "labels": [
          "Gene"
        ],
        "properties": {
          "license": "CC0 1.0",
          "identifier": {
            "low": 1616,
            "high": 0
          },
          "chromosome": "6",
          "name": "DAXX",
          "description": "death-domain associated protein",
          "source": "Entrez Gene",
          "url": "http://identifiers.org/ncbigene/1616"
        }
      },
      {
        "identity": {
          "low": 560370,
          "high": 0
        },
        "start": {
          "low": 26360,
          "high": 0
        },
        "end": {
```

```
          "low": 1204,
          "high": 0
        },
        "type": "UPREGULATES_CuG",
        "properties": {
          "method": "measured",
          "unbiased": true,
          "z_score": 4.861,
          "source": "LINCS L1000"
        }
      }
    ],
    "_fieldLookup": {
      "source": 0,
      "target": 1,
      "relationship": 2
    }
  },
  {
    "keys": [
      "source",
      "target",
      "relationship"
    ],
    "length": 3,
    "_fields": [
      {
        "identity": {
          "low": 31332,
          "high": 0
        },
        "labels": [
          "Gene"
        ],
        "properties": {
          "license": "CC0 1.0",
          "identifier": {
            "low": 2104,
            "high": 0
          },
          "chromosome": "1",
          "name": "ESRRG",
          "description": "estrogen-related receptor gamma",
          "source": "Entrez Gene",
          "url": "http://identifiers.org/ncbigene/2104"
        }
      },
      {
        "identity": {
          "low": 14102,
          "high": 0
        },
        "labels": [
          "Gene"
        ],
        "properties": {
          "license": "CC0 1.0",
          "identifier": {
            "low": 4118,
            "high": 0
          },
```

```
        "chromosome": "2",
        "name": "MAL",
        "description": "mal, T-cell differentiation protein",
        "source": "Entrez Gene",
        "url": "http://identifiers.org/ncbigene/4118"
      }
    },
    {
      "identity": {
        "low": 1046694,
        "high": 0
      },
      "start": {
        "low": 31332,
        "high": 0
      },
      "end": {
        "low": 14102,
        "high": 0
      },
      "type": "REGULATES_GrG",
      "properties": {
        "method": "imputed",
        "unbiased": true,
        "source": "LINCS L1000",
        "subtypes": [
          "overexpression upregulates"
        ]
      }
    }
  ],
  "_fieldLookup": {
    "source": 0,
    "target": 1,
    "relationship": 2
  }
},
{
  "keys": [
    "source",
    "target",
    "relationship"
  ],
  "length": 3,
  "_fields": [
    {
      "identity": {
        "low": 26360,
        "high": 0
      },
      "labels": [
        "Compound"
      ],
      "properties": {
        "license": "CC BY-NC 4.0",
        "identifier": "DB00162",
        "inchikey": "InChIKey=FPIPGXGPPPQFEQ-OVSJKPMPSA-N",
        "inchi": "InChI=1S/C20H30O/c1-16(8-6-9-17(2)13-15-21)11-12-19-18(3)
        10-7-14-20(19,4)5/h6,8-9,11-13,21H,7,10,14-15H2,1-5H3/b9-6+,12-11+,
        16-8+,17-13+",
        "name": "Vitamin A",
```

```
          "source": "DrugBank",
          "url": "http://www.drugbank.ca/drugs/DB00162"
        }
      },
      {
        "identity": {
          "low": 14102,
          "high": 0
        },
        "labels": [
          "Gene"
        ],
        "properties": {
          "license": "CC0 1.0",
          "identifier": {
            "low": 4118,
            "high": 0
          },
          "chromosome": "2",
          "name": "MAL",
          "description": "mal, T-cell differentiation protein",
          "source": "Entrez Gene",
          "url": "http://identifiers.org/ncbigene/4118"
        }
      },
      {
        "identity": {
          "low": 703219,
          "high": 0
        },
        "start": {
          "low": 26360,
          "high": 0
        },
        "end": {
          "low": 14102,
          "high": 0
        },
        "type": "UPREGULATES_CuG",
        "properties": {
          "method": "imputed",
          "unbiased": true,
          "z_score": 4.714,
          "source": "LINCS L1000"
        }
      }
    ],
    "_fieldLookup": {
      "source": 0,
      "target": 1,
      "relationship": 2
    }
  },
  {
    "keys": [
      "source",
      "target",
      "relationship"
    ],
    "length": 3,
    "_fields": [
```

```
{
  "identity": {
    "low": 31332,
    "high": 0
  },
  "labels": [
    "Gene"
  ],
  "properties": {
    "license": "CC0 1.0",
    "identifier": {
      "low": 2104,
      "high": 0
    },
    "chromosome": "1",
    "name": "ESRRG",
    "description": "estrogen-related receptor gamma",
    "source": "Entrez Gene",
    "url": "http://identifiers.org/ncbigene/2104"
  }
},
{
  "identity": {
    "low": 19319,
    "high": 0
  },
  "labels": [
    "Gene"
  ],
  "properties": {
    "license": "CC0 1.0",
    "identifier": {
      "low": 5898,
      "high": 0
    },
    "chromosome": "7",
    "name": "RALA",
    "description": "v-ral simian leukemia viral oncogene homolog A
    (ras related)",
    "source": "Entrez Gene",
    "url": "http://identifiers.org/ncbigene/5898"
  }
},
{
  "identity": {
    "low": 940051,
    "high": 0
  },
  "start": {
    "low": 31332,
    "high": 0
  },
  "end": {
    "low": 19319,
    "high": 0
  },
  "type": "REGULATES_GrG",
  "properties": {
    "method": "measured",
    "unbiased": true,
    "source": "LINCS L1000",
```

```
          "subtypes": [
            "overexpression upregulates"
          ]
        }
      }
    ],
    "_fieldLookup": {
      "source": 0,
      "target": 1,
      "relationship": 2
    }
  },
  {
    "keys": [
      "source",
      "target",
      "relationship"
    ],
    "length": 3,
    "_fields": [
      {
        "identity": {
          "low": 26360,
          "high": 0
        },
        "labels": [
          "Compound"
        ],
        "properties": {
          "license": "CC BY-NC 4.0",
          "identifier": "DB00162",
          "inchikey": "InChIKey=FPIPGXGPPPQFEQ-OVSJKPMPSA-N",
          "inchi": "InChI=1S/C20H30O/c1-16(8-6-9-17(2)13-15-21)11-12-19-18(3)
          10-7-14-20(19,4)5/h6,8-9,11-13,21H,7,10,14-15H2,1-5H3/b9-6+,12-11+,
          16-8+,17-13+",
          "name": "Vitamin A",
          "source": "DrugBank",
          "url": "http://www.drugbank.ca/drugs/DB00162"
        }
      },
      {
        "identity": {
          "low": 19319,
          "high": 0
        },
        "labels": [
          "Gene"
        ],
        "properties": {
          "license": "CC0 1.0",
          "identifier": {
            "low": 5898,
            "high": 0
          },
          "chromosome": "7",
          "name": "RALA",
          "description": "v-ral simian leukemia viral oncogene homolog A
          (ras related)",
          "source": "Entrez Gene",
          "url": "http://identifiers.org/ncbigene/5898"
        }
```

```
      },
      {
        "identity": {
          "low": 867114,
          "high": 0
        },
        "start": {
          "low": 26360,
          "high": 0
        },
        "end": {
          "low": 19319,
          "high": 0
        },
        "type": "DOWNREGULATES_CdG",
        "properties": {
          "method": "measured",
          "unbiased": true,
          "z_score": -4.186,
          "source": "LINCS L1000"
        }
      }
    ],
    "_fieldLookup": {
      "source": 0,
      "target": 1,
      "relationship": 2
    }
  },
  {
    "keys": [
      "source",
      "target",
      "relationship"
    ],
    "length": 3,
    "_fields": [
      {
        "identity": {
          "low": 31332,
          "high": 0
        },
        "labels": [
          "Gene"
        ],
        "properties": {
          "license": "CC0 1.0",
          "identifier": {
            "low": 2104,
            "high": 0
          },
          "chromosome": "1",
          "name": "ESRRG",
          "description": "estrogen-related receptor gamma",
          "source": "Entrez Gene",
          "url": "http://identifiers.org/ncbigene/2104"
        }
      },
      {
        "identity": {
          "low": 20458,
```

```
          "high": 0
        },
        "labels": [
          "Gene"
        ],
        "properties": {
          "license": "CC0 1.0",
          "identifier": {
            "low": 291,
            "high": 0
          },
          "chromosome": "4",
          "name": "SLC25A4",
          "description": "solute carrier family 25 (mitochondrial carrier;
          adenine nucleotide translocator), member 4",
          "source": "Entrez Gene",
          "url": "http://identifiers.org/ncbigene/291"
        }
      },
      {
        "identity": {
          "low": 175482,
          "high": 0
        },
        "start": {
          "low": 31332,
          "high": 0
        },
        "end": {
          "low": 20458,
          "high": 0
        },
        "type": "REGULATES_GrG",
        "properties": {
          "method": "measured",
          "unbiased": true,
          "source": "LINCS L1000",
          "subtypes": [
            "overexpression upregulates"
          ]
        }
      }
    ],
    "_fieldLookup": {
      "source": 0,
      "target": 1,
      "relationship": 2
    }
  },
  {
    "keys": [
      "source",
      "target",
      "relationship"
    ],
    "length": 3,
    "_fields": [
      {
        "identity": {
          "low": 26360,
          "high": 0
```

```
      },
      "labels": [
        "Compound"
      ],
      "properties": {
        "license": "CC BY-NC 4.0",
        "identifier": "DB00162",
        "inchikey": "InChIKey=FPIPGXGPPPQFEQ-OVSJKPMPSA-N",
        "inchi": "InChI=1S/C20H30O/c1-16(8-6-9-17(2)13-15-21)11-12-19-18(3)
        10-7-14-20(19,4)5/h6,8-9,11-13,21H,7,10,14-15H2,1-5H3/b9-6+,12-11+,
        16-8+,17-13+",
        "name": "Vitamin A",
        "source": "DrugBank",
        "url": "http://www.drugbank.ca/drugs/DB00162"
      }
    },
    {
      "identity": {
        "low": 20458,
        "high": 0
      },
      "labels": [
        "Gene"
      ],
      "properties": {
        "license": "CC0 1.0",
        "identifier": {
          "low": 291,
          "high": 0
        },
        "chromosome": "4",
        "name": "SLC25A4",
        "description": "solute carrier family 25 (mitochondrial carrier;
        adenine nucleotide translocator), member 4",
        "source": "Entrez Gene",
        "url": "http://identifiers.org/ncbigene/291"
      }
    },
    {
      "identity": {
        "low": 281339,
        "high": 0
      },
      "start": {
        "low": 26360,
        "high": 0
      },
      "end": {
        "low": 20458,
        "high": 0
      },
      "type": "DOWNREGULATES_CdG",
      "properties": {
        "method": "measured",
        "unbiased": true,
        "z_score": -4.541,
        "source": "LINCS L1000"
      }
    }
  ],
  "_fieldLookup": {
```

```
      "source": 0,
      "target": 1,
      "relationship": 2
    }
  },
  {
    "keys": [
      "source",
      "target",
      "relationship"
    ],
    "length": 3,
    "_fields": [
      {
        "identity": {
          "low": 31332,
          "high": 0
        },
        "labels": [
          "Gene"
        ],
        "properties": {
          "license": "CC0 1.0",
          "identifier": {
            "low": 2104,
            "high": 0
          },
          "chromosome": "1",
          "name": "ESRRG",
          "description": "estrogen-related receptor gamma",
          "source": "Entrez Gene",
          "url": "http://identifiers.org/ncbigene/2104"
        }
      },
      {
        "identity": {
          "low": 41548,
          "high": 0
        },
        "labels": [
          "Gene"
        ],
        "properties": {
          "license": "CC0 1.0",
          "identifier": {
            "low": 994,
            "high": 0
          },
          "chromosome": "20",
          "name": "CDC25B",
          "description": "cell division cycle 25B",
          "source": "Entrez Gene",
          "url": "http://identifiers.org/ncbigene/994"
        }
      },
      {
        "identity": {
          "low": 2086330,
          "high": 0
        },
        "start": {
```

```
          "low": 31332,
          "high": 0
        },
        "end": {
          "low": 41548,
          "high": 0
        },
        "type": "REGULATES_GrG",
        "properties": {
          "method": "measured",
          "unbiased": true,
          "source": "LINCS L1000",
          "subtypes": [
            "overexpression downregulates"
          ]
        }
      }
    ],
    "_fieldLookup": {
      "source": 0,
      "target": 1,
      "relationship": 2
    }
  },
  {
    "keys": [
      "source",
      "target",
      "relationship"
    ],
    "length": 3,
    "_fields": [
      {
        "identity": {
          "low": 26360,
          "high": 0
        },
        "labels": [
          "Compound"
        ],
        "properties": {
          "license": "CC BY-NC 4.0",
          "identifier": "DB00162",
          "inchikey": "InChIKey=FPIPGXGPPPQFEQ-OVSJKPMPSA-N",
          "inchi": "InChI=1S/C20H30O/c1-16(8-6-9-17(2)13-15-21)11-12-19-18
          (3)10-7-14-20(19,4)5/h6,8-9,11-13,21H,7,10,14-15H2,1-5H3/b9-6+,
          12-11+,16-8+,17-13+",
          "name": "Vitamin A",
          "source": "DrugBank",
          "url": "http://www.drugbank.ca/drugs/DB00162"
        }
      },
      {
        "identity": {
          "low": 41548,
          "high": 0
        },
        "labels": [
          "Gene"
        ],
        "properties": {
```

```
          "license": "CC0 1.0",
          "identifier": {
            "low": 994,
            "high": 0
          },
          "chromosome": "20",
          "name": "CDC25B",
          "description": "cell division cycle 25B",
          "source": "Entrez Gene",
          "url": "http://identifiers.org/ncbigene/994"
        }
      },
      {
        "identity": {
          "low": 2114139,
          "high": 0
        },
        "start": {
          "low": 26360,
          "high": 0
        },
        "end": {
          "low": 41548,
          "high": 0
        },
        "type": "DOWNREGULATES_CdG",
        "properties": {
          "method": "measured",
          "unbiased": true,
          "z_score": -4.652,
          "source": "LINCS L1000"
        }
      }
    ],
    "_fieldLookup": {
      "source": 0,
      "target": 1,
      "relationship": 2
    }
  },
  {
    "keys": [
      "source",
      "target",
      "relationship"
    ],
    "length": 3,
    "_fields": [
      {
        "identity": {
          "low": 39730,
          "high": 0
        },
        "labels": [
          "Anatomy"
        ],
        "properties": {
          "license": "CC BY 3.0",
          "identifier": "UBERON:0000033",
          "mesh_id": "D006257",
          "name": "head",
```

```
          "source": "Uberon",
          "bto_id": "BTO:0000282",
          "url": "http://purl.obolibrary.org/obo/UBERON_0000033"
        }
      },
      {
        "identity": {
          "low": 31332,
          "high": 0
        },
        "labels": [
          "Gene"
        ],
        "properties": {
          "license": "CC0 1.0",
          "identifier": {
            "low": 2104,
            "high": 0
          },
          "chromosome": "1",
          "name": "ESRRG",
          "description": "estrogen-related receptor gamma",
          "source": "Entrez Gene",
          "url": "http://identifiers.org/ncbigene/2104"
        }
      },
      {
        "identity": {
          "low": 457647,
          "high": 0
        },
        "start": {
          "low": 39730,
          "high": 0
        },
        "end": {
          "low": 31332,
          "high": 0
        },
        "type": "EXPRESSES_AeG",
        "properties": {
          "license": "CC BY 4.0",
          "sources": [
            "TISSUES"
          ],
          "unbiased": false
        }
      }
    ],
    "_fieldLookup": {
      "source": 0,
      "target": 1,
      "relationship": 2
    }
  },
  {
    "keys": [
      "source",
      "target",
      "relationship"
    ],
```

```
"length": 3,
"_fields": [
  {
    "identity": {
      "low": 20344,
      "high": 0
    },
    "labels": [
      "Disease"
    ],
    "properties": {
      "license": "CC BY 3.0",
      "identifier": "DOID:9296",
      "name": "cleft lip",
      "source": "Disease Ontology",
      "url": "http://purl.obolibrary.org/obo/DOID_9296"
    }
  },
  {
    "identity": {
      "low": 39730,
      "high": 0
    },
    "labels": [
      "Anatomy"
    ],
    "properties": {
      "license": "CC BY 3.0",
      "identifier": "UBERON:0000033",
      "mesh_id": "D006257",
      "name": "head",
      "source": "Uberon",
      "bto_id": "BTO:0000282",
      "url": "http://purl.obolibrary.org/obo/UBERON_0000033"
    }
  },
  {
    "identity": {
      "low": 1737730,
      "high": 0
    },
    "start": {
      "low": 20344,
      "high": 0
    },
    "end": {
      "low": 39730,
      "high": 0
    },
    "type": "LOCALIZES_DlA",
    "properties": {
      "license": "CC0 1.0",
      "unbiased": false,
      "source": "MEDLINE cooccurrence"
    }
  }
],
"_fieldLookup": {
  "source": 0,
  "target": 1,
  "relationship": 2
```

```
    }
  },
  {
    "keys": [
      "source",
      "target",
      "relationship"
    ],
    "length": 3,
    "_fields": [
      {
        "identity": {
          "low": 31394,
          "high": 0
        },
        "labels": [
          "Anatomy"
        ],
        "properties": {
          "license": "CC BY 3.0",
          "identifier": "UBERON:0001893",
          "mesh_id": "D013687",
          "name": "telencephalon",
          "source": "Uberon",
          "bto_id": "BTO:0000239",
          "url": "http://purl.obolibrary.org/obo/UBERON_0001893"
        }
      },
      {
        "identity": {
          "low": 31332,
          "high": 0
        },
        "labels": [
          "Gene"
        ],
        "properties": {
          "license": "CC0 1.0",
          "identifier": {
            "low": 2104,
            "high": 0
          },
          "chromosome": "1",
          "name": "ESRRG",
          "description": "estrogen-related receptor gamma",
          "source": "Entrez Gene",
          "url": "http://identifiers.org/ncbigene/2104"
        }
      },
      {
        "identity": {
          "low": 1813067,
          "high": 0
        },
        "start": {
          "low": 31394,
          "high": 0
        },
        "end": {
          "low": 31332,
          "high": 0
```

```
        },
        "type": "EXPRESSES_AeG",
        "properties": {
          "sources": [
            "Bgee"
          ],
          "unbiased": true
        }
      }
    ],
    "_fieldLookup": {
      "source": 0,
      "target": 1,
      "relationship": 2
    }
  },
  {
    "keys": [
      "source",
      "target",
      "relationship"
    ],
    "length": 3,
    "_fields": [
      {
        "identity": {
          "low": 20344,
          "high": 0
        },
        "labels": [
          "Disease"
        ],
        "properties": {
          "license": "CC BY 3.0",
          "identifier": "DOID:9296",
          "name": "cleft lip",
          "source": "Disease Ontology",
          "url": "http://purl.obolibrary.org/obo/DOID_9296"
        }
      },
      {
        "identity": {
          "low": 31394,
          "high": 0
        },
        "labels": [
          "Anatomy"
        ],
        "properties": {
          "license": "CC BY 3.0",
          "identifier": "UBERON:0001893",
          "mesh_id": "D013687",
          "name": "telencephalon",
          "source": "Uberon",
          "bto_id": "BTO:0000239",
          "url": "http://purl.obolibrary.org/obo/UBERON_0001893"
        }
      },
      {
        "identity": {
          "low": 930814,
```

```
          "high": 0
        },
        "start": {
          "low": 20344,
          "high": 0
        },
        "end": {
          "low": 31394,
          "high": 0
        },
        "type": "LOCALIZES_DlA",
        "properties": {
          "license": "CC0 1.0",
          "unbiased": false,
          "source": "MEDLINE cooccurrence"
        }
      }
    ],
    "_fieldLookup": {
      "source": 0,
      "target": 1,
      "relationship": 2
    }
  },
  {
    "keys": [
      "source",
      "target",
      "relationship"
    ],
    "length": 3,
    "_fields": [
      {
        "identity": {
          "low": 31332,
          "high": 0
        },
        "labels": [
          "Gene"
        ],
        "properties": {
          "license": "CC0 1.0",
          "identifier": {
            "low": 2104,
            "high": 0
          },
          "chromosome": "1",
          "name": "ESRRG",
          "description": "estrogen-related receptor gamma",
          "source": "Entrez Gene",
          "url": "http://identifiers.org/ncbigene/2104"
        }
      },
      {
        "identity": {
          "low": 28322,
          "high": 0
        },
        "labels": [
          "Gene"
        ],
```

```
        "properties": {
          "license": "CC0 1.0",
          "identifier": {
            "low": 57698,
            "high": 0
          },
          "chromosome": "10",
          "name": "SHTN1",
          "description": "shootin 1",
          "source": "Entrez Gene",
          "url": "http://identifiers.org/ncbigene/57698"
        }
      },
      {
        "identity": {
          "low": 2187437,
          "high": 0
        },
        "start": {
          "low": 31332,
          "high": 0
        },
        "end": {
          "low": 28322,
          "high": 0
        },
        "type": "INTERACTS_GiG",
        "properties": {
          "sources": [
            "HI-II-14",
            "II_binary"
          ],
          "unbiased": true
        }
      }
    ],
    "_fieldLookup": {
      "source": 0,
      "target": 1,
      "relationship": 2
    }
  },
  {
    "keys": [
      "source",
      "target",
      "relationship"
    ],
    "length": 3,
    "_fields": [
      {
        "identity": {
          "low": 20344,
          "high": 0
        },
        "labels": [
          "Disease"
        ],
        "properties": {
          "license": "CC BY 3.0",
          "identifier": "DOID:9296",
```

```
          "name": "cleft lip",
          "source": "Disease Ontology",
          "url": "http://purl.obolibrary.org/obo/DOID_9296"
        }
      },
      {
        "identity": {
          "low": 28322,
          "high": 0
        },
        "labels": [
          "Gene"
        ],
        "properties": {
          "license": "CC0 1.0",
          "identifier": {
            "low": 57698,
            "high": 0
          },
          "chromosome": "10",
          "name": "SHTN1",
          "description": "shootin 1",
          "source": "Entrez Gene",
          "url": "http://identifiers.org/ncbigene/57698"
        }
      },
      {
        "identity": {
          "low": 1413702,
          "high": 0
        },
        "start": {
          "low": 20344,
          "high": 0
        },
        "end": {
          "low": 28322,
          "high": 0
        },
        "type": "ASSOCIATES_DaG",
        "properties": {
          "license": "CC BY 4.0",
          "sources": [
            "GWAS Catalog"
          ],
          "unbiased": true
        }
      }
    ],
    "_fieldLookup": {
      "source": 0,
      "target": 1,
      "relationship": 2
    }
  },
  {
    "keys": [
      "source",
      "target",
      "relationship"
    ],
```

```
"length": 3,
"_fields": [
  {
    "identity": {
      "low": 15219,
      "high": 0
    },
    "labels": [
      "Anatomy"
    ],
    "properties": {
      "license": "CC BY 3.0",
      "identifier": "UBERON:0000922",
      "mesh_id": "D004622",
      "name": "embryo",
      "source": "Uberon",
      "bto_id": "BTO:0000379",
      "url": "http://purl.obolibrary.org/obo/UBERON_0000922"
    }
  },
  {
    "identity": {
      "low": 31332,
      "high": 0
    },
    "labels": [
      "Gene"
    ],
    "properties": {
      "license": "CC0 1.0",
      "identifier": {
        "low": 2104,
        "high": 0
      },
      "chromosome": "1",
      "name": "ESRRG",
      "description": "estrogen-related receptor gamma",
      "source": "Entrez Gene",
      "url": "http://identifiers.org/ncbigene/2104"
    }
  },
  {
    "identity": {
      "low": 2106094,
      "high": 0
    },
    "start": {
      "low": 15219,
      "high": 0
    },
    "end": {
      "low": 31332,
      "high": 0
    },
    "type": "EXPRESSES_AeG",
    "properties": {
      "license": "CC BY 4.0",
      "sources": [
        "TISSUES"
      ],
      "unbiased": false
```

```
        }
      }
    ],
    "_fieldLookup": {
      "source": 0,
      "target": 1,
      "relationship": 2
    }
  },
  {
    "keys": [
      "source",
      "target",
      "relationship"
    ],
    "length": 3,
    "_fields": [
      {
        "identity": {
          "low": 20344,
          "high": 0
        },
        "labels": [
          "Disease"
        ],
        "properties": {
          "license": "CC BY 3.0",
          "identifier": "DOID:9296",
          "name": "cleft lip",
          "source": "Disease Ontology",
          "url": "http://purl.obolibrary.org/obo/DOID_9296"
        }
      },
      {
        "identity": {
          "low": 15219,
          "high": 0
        },
        "labels": [
          "Anatomy"
        ],
        "properties": {
          "license": "CC BY 3.0",
          "identifier": "UBERON:0000922",
          "mesh_id": "D004622",
          "name": "embryo",
          "source": "Uberon",
          "bto_id": "BTO:0000379",
          "url": "http://purl.obolibrary.org/obo/UBERON_0000922"
        }
      },
      {
        "identity": {
          "low": 1167611,
          "high": 0
        },
        "start": {
          "low": 20344,
          "high": 0
        },
        "end": {
```

```
          "low": 15219,
          "high": 0
        },
        "type": "LOCALIZES_DlA",
        "properties": {
          "license": "CC0 1.0",
          "unbiased": false,
          "source": "MEDLINE cooccurrence"
        }
      }
    ],
    "_fieldLookup": {
      "source": 0,
      "target": 1,
      "relationship": 2
    }
  }
]
```
